# Stable, Precise, and Reproducible Patterning of Bicoid and Hunchback Molecules in the Early *Drosophila* Embryo

**DOI:** 10.1371/journal.pcbi.1000486

**Published:** 2009-08-28

**Authors:** Yurie Okabe-Oho, Hiroki Murakami, Suguru Oho, Masaki Sasai

**Affiliations:** 1 Department of Computational Science and Engineering, Nagoya University, Nagoya, Japan; 2 Department of Environmental Engineering and Architecture, Nagoya University, Nagoya, Japan; 3 Department of Applied Physics, Nagoya University, Nagoya, Japan; 4 School of Computational Sciences, Korea Institute for Advanced Study, Seoul, Korea; Medical College of Wisconsin, United States of America

## Abstract

Precise patterning of morphogen molecules and their accurate reading out are of key importance in embryonic development. Recent experiments have visualized distributions of proteins in developing embryos and shown that the gradient of concentration of Bicoid morphogen in *Drosophila* embryos is established rapidly after fertilization and remains stable through syncytial mitoses. This stable Bicoid gradient is read out in a precise way to distribute Hunchback with small fluctuations in each embryo and in a reproducible way, with small embryo-to-embryo fluctuation. The mechanisms of such stable, precise, and reproducible patterning through noisy cellular processes, however, still remain mysterious. To address these issues, here we develop the one- and three-dimensional stochastic models of the early *Drosophila* embryo. The simulated results show that the fluctuation in expression of the *hunchback* gene is dominated by the random arrival of Bicoid at the *hunchback* enhancer. Slow diffusion of Hunchback protein, however, averages out this intense fluctuation, leading to the precise patterning of distribution of Hunchback without loss of sharpness of the boundary of its distribution. The coordinated rates of diffusion and transport of input Bicoid and output Hunchback play decisive roles in suppressing fluctuations arising from the dynamical structure change in embryos and those arising from the random diffusion of molecules, and give rise to the stable, precise, and reproducible patterning of Bicoid and Hunchback distributions.

## Author Summary

For developing embryos, the precise, position-specific regulation of molecular processes is of fatal importance. As the mechanism of such regulation, widely accepted has been the notion of the intraembryonic distribution of regulatory molecules called “morphogens”. One of the best-studied morphogens is Bicoid in the early developmental stage of the *Drosophila* embryo. Synthesized around the anterior pole of the embryo, Bicoid forms an exponential gradient of concentration to initiate expression of a target gene, *hunchback*, in nuclei at the periphery of the embryo. This invariably forms a concentration boundary of the product protein Hunchback at around 49% embryo length. Remarkably, the embryo-embryo variability in the boundary position is less than 5%. Reactions in embryos, however, should be intrinsically noisy because the number of molecules involved is small, and those reactions are governed by randomly diffusing molecules. The mechanisms to generate the invariable Hunchback distribution by filtering the intense noise remain mysterious, and here we construct models to shed light on this problem. Stochastic simulations show that the slow diffusion of Hunchback averages out the intense noise, so that the coordinated rates of diffusion and transport of input Bicoid and output Hunchback play decisive roles in suppressing fluctuations.

## Introduction

Pattern formation of multicellular organisms requires the accurate spatial regulation of gene expression. Such regulation has been attributed to the position dependent distribution of signaling molecules called morphogens [1],[2]. One of the best studied morphogens is Bicoid (Bcd) in embryos of *Drosophila melanogaster*, which works as a transcription factor to regulate the expression of *hunchback* (*hb*) and other downstream genes [3]–[5]. Bcd is synthesized from the maternal mRNA which is localized at around the anterior pole of embryo, and the concentration of Bcd exhibits a gradient with approximately exponential decay with distance from the anterior pole [6]. Although the decisive importance of the gradient in concentration of Bcd has been established [7],[8], the mechanism for forming the stable Bcd gradient and that for the reliable readout of the gradient remain largely unresolved.

Important advances have been made by visualizing spatial patterns of distributions of the input protein Bcd and the output protein Hunchback (Hb) with the antibody staining technique [6],[9],[10]. These visualized data, however, have been taken from fixed embryos, which provided only the snapshots of developing embryos. Further insights were gained by combining the method using the enhanced green fluorescent protein (eGFP) with the antibody staining technique: In two papers [11],[12], Gregor et al. reported the data of dynamically changing Bcd patterns in living embryos by observing fluorescence of a Bcd-eGFP fusion protein. These data provoked fundamental questions as to the formation and readout of the Bcd gradient, which presents challenges in computational biology.

In their first paper [11], Gregor et al. showed that the Bcd distribution is approximately an exponential gradient in intranuclear concentration, which is established rather rapidly in less than 90 min after fertilization. As Bcd is a DNA-binding protein, Bcd is localized in nuclei. However, by observing responses of distributions of Bcd-eGFP to the photo-bleaching stimuli, it was shown that Bcd is not simply trapped in nuclei but is in dynamic equilibrium between influx and efflux with the cytoplasm and intranuclear degradation. The early embryo in the blastodermal stage undergoes a series of syncytial mitoses from nuclear cycles 10 to 14, during which the nuclei are not separated by cell membranes. It was observed that Bcd which is stored in nuclei is released into cytoplasm as nuclei lose their envelopes during each mitosis, and the released Bcd is concentrated into nuclei again as envelopes are reformed during the interphase period. These observations raised the question on how the stable Bcd gradient is established in spite of the repeated dynamical structural change of embryo due to mitoses of nuclei. Though the effects of dynamically changing nuclei have begun to be to be investigated theoretically [13]–[15], most of the hitherto developed theoretical models of the Bcd gradient have been based on the assumptions of diffusion and degradation of Bcd in a static continuum [16]–[20].

In the second paper [12], the authors reported the data of fluctuations in concentration profiles of Bcd and Hb and showed that formation of those profiles is precise and reproducible: It was shown that fluctuations of profiles of Bcd and Hb in each embryo are about 10%, which are small enough to distinguish adjacent nuclei, and that fluctuations from embryo to embryo are also as small as 10%. These data imply that the system exerts precise control over concentration of Bcd, and that Hb is produced as a reliable response to the small concentration difference of Bcd. Response of the *hb* activity to Bcd, however, is the process to receive randomly arriving Bcd molecules at the *hb* enhancer on DNA. The random arrival of Bcd at the nanometer scale region of DNA should induce intense noise in the *hb* activity and Gregor et al. argued that this effect should be so large that we have to assume some unconfirmed mechanism working to reduce the noise of the readout process to the observed level of precision. There are further sources of intrinsic noise in the readout process arising from the bursting production of proteins and the nonadiabatic gene switching [21]–[28], so that the observed precise readout of the Bcd gradient implies that the noise arising from those sources is also suppressed to the level of 10% fluctuations.

In this paper, we construct two computational models, one-dimensional and three-dimensional models of *Drosophila* embryo, to discuss two major issues raised by Gregor et al.; how the stable patterning of the Bcd gradient is realized in the dynamically changing syncytial embryo; and how the Bcd gradient is read in a precise and reproducible way in spite of intense noise induced by the random diffusion of Bcd molecules. Our one-dimensional model of embryo focuses on the latter issue on the noise induced by random diffusion, and effects of the dynamically changing embryonic structure are examined with the three-dimensional model.

Importance of the noise induced by random diffusion has been stressed by Berg and Purcell in their early study of bacterial chemotaxis [29]. They have argued that certain physical limit of sensitivity should exist when the bacterial receptor receives the randomly arriving chemical signals. Bialek and colleagues [30]–[32] extended this analysis in a theoretically more systematic way and showed that the physical limit arising from random diffusion of signaling molecules is not the special feature of bacterial chemotaxis, but is common to many biological processes of receiving chemical signals. Gregor et al. [12] applied Bialek's theory to the problem of *Drosophila* embryo and showed that the limit of accuracy in sensing the Bcd concentration is
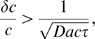
(1)where *c* is the intranuclear concentration of Bcd, and *δc* is fluctuation in the Bcd concentration sensed by the *hb* enhancer. The *hb* enhancer is assumed to have the typical length *a*. *D* is the intranuclear diffusion constant of Bcd, and *τ* is the time length during which Bcd can bind to the enhancer. In *Drosophila* embryo, decision of whether *hb* is activated or not is made at the point of threshold concentration 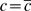; *hb* is activated in the nuclei of 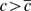 and is kept silent if 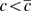. Since the observed values of *D* and 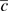 are 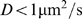 and 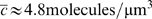 [12], and the size of the enhancer binding site should be as small as 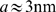, Equation 1 implies 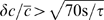 at the decision point of the *hb* activation [12]. When we regard *τ* as the typical time length of one nuclear cycle, we have 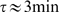, which leads to 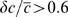. In Ref.12, *δc* was estimated from the data of fluctuation of the Hb concentration by assuming that *δc* is reflected to the *hb* gene activity and hence is reflected to the fluctuation in concentration of Hb. The value observed in this way was 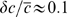, which is much smaller than the expected value of 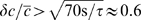. To explain the small value of 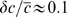, *τ* should be almost two hours, which is unacceptably long and near to the entire time available for development from fertilization up to cellularization. Thus, some mechanism lacking in this argument is needed to reduce the expected large noise of 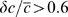 to the level of the observed precision 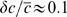. We may refer to this problem as *the paradox of signal interpretation*.

**Figure 1:**
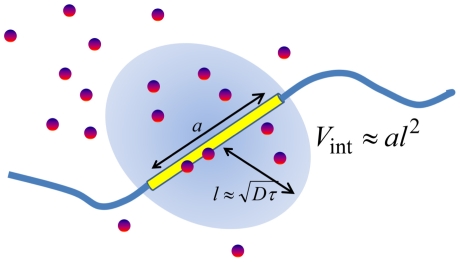
Interaction volume. Effects of the random arrival of Bcd at the *hb* enhancer on DNA can be intuitively explained with the concept of interaction volume. Since molecules diffuse over a distance 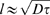 during the allowed time *τ*, Bcd molecules which can interact with the target of size *a* during the period *τ* are distributed in the volume of size 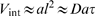, which is called the interaction volume. The interaction volume is small when *a* is in a nanometer length-scale. With the average Bcd concentration *c*, the average number of Bcd molecules in the interaction volume is 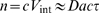. If Bcd molecules diffuse as independent particles, fluctuation in *n* should obey the Poisson statistics to exhibit 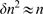. Then, the Bcd concentration sensed at the target fluctuates to the extent at least 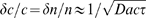. Smallness of 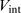 leads to the large 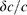.

Though Bialek and colleagues derived Equation 1 by solving the coupled equations of diffusion, degradation, and binding/unbinding processes, the same result can be obtained by the intuitive argument as illustrated in Figure 1: Since Bcd molecules diffuse over a distance 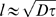 during the allowed time *τ*, Bcd molecules interacting with the target of size *a* during the period *τ* are distributed in the volume 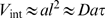. We call this volume “interaction volume”. The number of molecules in the interaction volume is 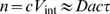 in average. If Bcd molecules diffuse as independent particles, fluctuation in *n* should obey the Poisson statistics to exhibit 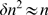. Then, we have 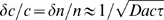, which gives the lower limit of fluctuation, and in more generic cases with other additional sources of noise, we have the criterion of Equation 1. In the following, we explicitly use the idea of interaction volume to model the readout process in embryo.

In the next section, we first discuss the one-dimensional stochastic model of embryo. To focus on the problem of the paradox of signal interpretation, we neglect the dynamical structural change of embryo and assume that nuclei stay still without exhibiting nuclear cycles. With this model we perform the stochastic simulation of reactions and diffusion of Bcd and Hb. Bcd molecules are produced at the anterior pole, diffuse, degrade, and bind/unbind to/from the *hb* enhancer, and Hb molecules are produced with the rate depending on the *hb* enhancer status, and diffuse and degrade. With this one-dimensional model, fluctuations in distributions of Bcd and Hb can be examined in a wide range of parameters and we show that the self-averaging process due to the Hb diffusion should resolve the paradox of signal interpretation.

Effects of the dynamically changing embryonic structure are investigated by constructing a more realistic three-dimensional stochastic model of embryo. In the three-dimensional model, an embryo is represented by a cylinder consisting of 43,750 hexagonal sites. Bcd molecules are synthesized at the most front slice of this cylinder. The inside structure of this cylinder is modified during simulation by following the rules governing nuclear mitoses in the model: At the initial instance, one site at the core of the cylinder is assumed to be a nucleus. This nucleus starts to divide, and at the nuclear cycle 8–10, the multiplicated nuclei move to the peripheral. At the nuclear cycle 14, about 6000 sites at the surface layer of the cylinder are nuclear sites. Within the nuclear site the same reactions are assumed as in the one-dimensional model, and in other cytosolic sites degradation and diffusion of Bcd and Hb are considered. We show that patterning of the Bcd profile is stable and precise in spite of dynamic mitoses of nuclei in embryo. We further confirm that the same mechanism of self-averaging of Hb molecules as discussed with the one-dimensional model also works in the three-dimensional model to resolve the paradox of signal interpretation.

## Results

### One-dimensional model of *Drosophila* embryo

#### The one-dimensional system

**Figure 2:**
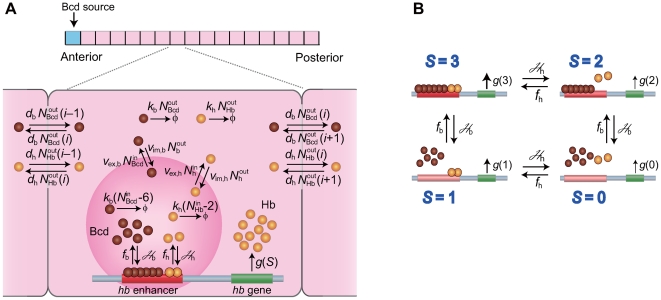
One-dimensional model of *Drosophila* embryo. (A) The one-dimensional system of length *L* is composed of 100 sites each of which has the length of Δ*x* = *L*/100. In each site diffusion and reactions of Bcd molecules (brown spheres) and Hb molecules (yellow spheres) are defined. Bcd diffuses from or into adjacent sites, degrades, and diffuses from or into the small region of interaction volume (dark pink, whose relative size is enlarged in the figure for explanation but its volume is about 1/1000 of nuclear volume). Hb is synthesized at each site and diffuses from or into adjacent sites, degrades, and diffuses from or into the small region of interaction volume. Bcd is synthesized only at the most anterior site. The rate of each process is written on each arrow. (B) The rate of Hb synthesis *g*(*S*) depends on the state of the *hb* enhancer, *S* = 0–3; the state with no bound Bcd or Hb (*S* = 0), the state with two bound Hb molecules and with no bound Bcd (*S* = 1), the state with six bound Bcd molecules and with no bound Hb (*S* = 2), and the state with six bound Bcd and two bound Hb molecules (*S* = 3). *g*(*S*) satisfies Equation 7 or Equation 8.

A simplified one-dimensional model of embryo is developed to focus on the problem of precise readout of the Bcd gradient. In this model, nuclei are assumed to stay static to simulate the stable distribution of Bcd during the interphase period of nuclear cycle 14. In this period, about 6000 nuclei are located in the vicinity of the surface of the embryo whose size varies around 500 µm [33]. Each of these nuclei is not wrapped by the cell membrane but is exposed to the common cytoplasm in a syncytial embryo. These nuclei are arranged in about 100 rows along the anterior-posterior (AP) axis of embryo [34]. Here, such a distribution of nuclei is modeled by the array of nuclei placed along the one-dimensional AP axis. As shown in Figure 2A, the one-dimensional system of length *L* is composed of *N* sites each of which has the length of Δ*x* = *L*/*N* with 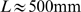 and *N* = 100. Each site contains a nucleus, and numbers of Bcd and Hb molecules in the *i*th site are denoted by *N*_Bcd_(*i*) and *N*_Hb_(*i*), respectively. With this system, we performed the stochastic simulation of the following molecular processes: (i) binding/unbinding of Bcd and Hb to/from the *hb* enhancer; (ii) diffusion and degradation; and (iii) synthesis of Bcd and Hb. See Text S1 for more detailed explanation of the model.

**(i) Binding/unbinding to/from the** ***hb*** **enhancer**: Random binding of diffusive molecules to the *hb* enhancer is the source of noise which should lead to the signal interpretation paradox. Since the size of the *hb* enhancer is in nanometer scale and a nucleus extends to the micrometer scale, each nucleus should be represented by the mesh of 1000 or more refined lattice points to directly describe this diffusion-binding process in the model. Such refinement requires the high computational cost, which should impair the merit of the one-dimensional model in survey of widely different parameter regions. Instead of refining the mesh size, we here use the concept of “interaction volume” to describe the random arrival of molecules at the nanometer region. We assume that Bcd and Hb have the same diffusion constant inside the nucleus and define the interaction volume of size 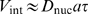, where 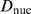 is the intra-nuclear diffusion constant of Bcd or Hb, *a* is the size of the *hb* enhancer, and *τ* is the time length of the interphase of nucleus. Use of different intra-nuclear diffusion constants for Bcd and Hb does not add significant effects in the present simplified model.

*N*_Bcd_(*i*) is divided into the part outside the interaction volume 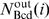, and the part inside the interaction volume 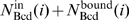, where 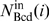 is the number of Bcd molecules that remain in the interaction volume without binding to the *hb* enhancer, and 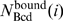 is the number of Bcd molecules that are bound to the *hb* enhancer. *N*_Hb_(*i*) is also divided in the same way, so that
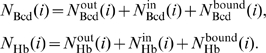
(2)We assume that only the molecules in the interaction volume can bind to the *hb* enhancer. Due to the intra-nuclear diffusion, molecules are stochastically transferred into/from the interaction volume. We write the volume of a nucleus as 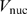, so that
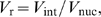
(3)represents the relative size of the interaction volume. The limit of *V*_r_ = 1 corresponds to the case that the inside of nucleus is so well stirred that the noise induced by the random arrival of Bcd at the *hb* enhancer is negligible. The smaller *V*_r_ is, the smaller 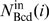 is allowed to stay in the interaction volume and fluctuations of 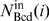 become larger, which enhances the noise in the binding reaction of Bcd. For *V*_r_≪1, the noise in the *hb* expression is dominated by this effect.

The *hb* enhancer contains multiple binding sites of Bcd and Hb, which should allow the various different states of the *hb* enhancer depending on the number of bound Bcd and Hb. As suggested in experiment [35],[36], we assume that the *hb* enhancer has six sites to bind Bcd and two sites to bind Hb. Since there can be cooperative interactions among bound Bcd and Hb [35]–[38], the most populated states should be categorized into the following four states; the state with no bound Bcd or Hb (*S*(*i*) = 0), the state with two bound Hb molecules and with no bound Bcd (*S*(*i*) = 1), the state with six bound Bcd molecules and with no bound Hb (*S*(*i*) = 2), and the state with six bound Bcd and two bound Hb molecules (*S*(*i*) = 3). We consider that the cooperative binding process of Bcd takes place as
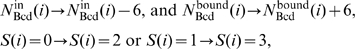
(4)with the rate 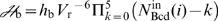 and that the cooperative binding process of Hb takes place as
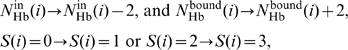
(5)with the rate 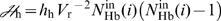. Due to the large fluctuation of 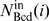 and 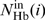, binding processes of Equation 4 and Equation 5 should bear intense noise as argued in the paradox of signal interpretation. In this paper, embryos homozygous for the mutated *hb* allele [36],[39],[40], which codes for Hb having no-DNA binding affinity, are also simulated by assuming reactions of Equation 4. For such mutant embryos, we put *h*_h_ = 0 and only consider *S*(*i*) = 0 and *S*(*i*) = 2 as possible states of the *hb* enhancer.

Unbinding reactions are the reverse processes of Equations 4 and 5 taking place with the rate 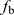 for the unbinding of Bcd and with the rate 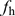 for the unbinding of Hb. The ratio 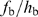 or 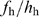 determines the affinity of Bcd or Hb to the *hb* enhancer site. We should also note that 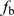 and 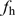 can be used as measures of time scale of change in the *hb* enhancer state.

**(ii) Diffusion and degradation**: Due to the diffusive movement in cytoplasm, Bcd molecules are transferred between adjacent sites with the rate *d*_b_ *N*_Bcd_(*i*), where *d*_b_ is proportional to the diffusion constant of Bcd, *D*_b_, as *d*_b_ = *D*_b_/Δ*x* ^2^. Bcd is degraded at each site with the rate 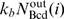 or 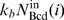. Diffusion and degradation of Hb are treated in the same way as those of Bcd but with different rate constants: Hb molecules are transferred between adjacent sites with the rate *d*_h_ *N*_Hb_(*i*), where *d*_h_ is proportional to the diffusion constant *D*_h_ as *d*_h_ = *D*_h_/Δ*x* ^2^. Hb is degraded at each site with the rate 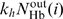 or 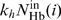.

**(iii) Synthesis:** Bcd is synthesized from the maternal mRNA distributed at around the anterior pole, which is simulated by assuming that Bcd is generated only at the site *i* = 1 with the rate *J*. Reactions involved in transcription and translation of *hb* are coarse-grained and described as a combined single reaction step, which is the approximation adopted in studies of gene expression [26]–[28]. Then, the process to synthesize Hb around the *i*th nucleus is
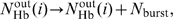
(6)where *N*_burst_ is the number of Hb molecules synthesized from a short-lived *hb* mRNA, representing the bursting production of Hb [25],[28]. The bursting production with *N*_burst_≫1 can be a source of intrinsic noise of *hb* expression [22],[28]. As illustrated in Figure 2B, we consider that Equation 6 is executed with the rate *g*(*S*(*i*)), where *S*(*i*) = 0–3 is the state of the *hb* enhancer in the *i*th site. As suggested by the experimental data [36],[40], binding of Hb and binding of Bcd should cooperatively activate synthesis of Hb, and hence we assume
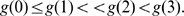
(7)For a mutant of loosing binding affinity of Hb with *h*_h_ = 0, we only consider the case of *S* = 0 and *S* = 2, so that
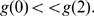
(8)The scheme of Bcd-Hb cooperation of Equation 7 implies that Hb activates expression of *hb*, which constitutes a positive feedback loop of Hb production. In the scheme of Equation 8, on the other hand, the positive feedback loop is absent. In this paper we compare two schemes of Equation 7 and Equation 8 to examine the effect of the positive feedback.

#### Simulation with the one-dimensional model

In the ensemble of many embryos, the average system size is approximately 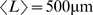, and *L* fluctuates from embryo to embryo with the relative amplitude of 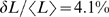 [12] where 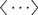 is the average over many embryos. Corresponding to these average and fluctuation, we repeat stochastic simulations 100 times to take the ensemble average of patterning of Bcd and Hb by randomly varying Δ*x* at each simulation run with 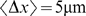, and 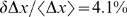. To analyze the calculated results of different trajectories in a unified way, 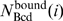, 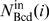, 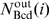, 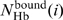, 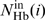, 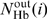, 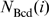, and 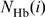, obtained at the last step of each simulation run are normalized by the factor 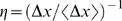 to be 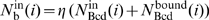, 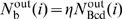, 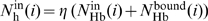, 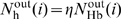, 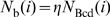, and 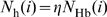. We consider that the intra-nuclear diffusion constant is similar to the one for the short-length diffusion in cytoplasm as *D*_nuc_ = 0.3–1.0 µm^2^/s [11]. Since *a* should be a typical length in DNA and *τ* should be a typical duration of interphase, we assume 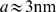 as the length of 10 base pairs and 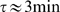, which leads to 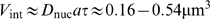. Diameter of a nucleus at nuclear cycle 14 is about 6.5 µm in average, so that 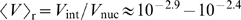. We use 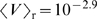.

Other parameters used in the model are explained in *Method* section and are summarized in Table 1. We refer to the parameterization of Table 1 as the standard parameterization in the one-dimensional model. Results of different parameter values from those of Table 1 are also examined extensively with the one-dimensional model. Especially to be mentioned in Table 1 is the value of the diffusion constant *D*_b_ of Bcd in cytoplasm. In the standard parameterization, we assume *D*_b_ = 10 µm^2^/s from the following two reasons: (i) The diffusion constant of inert molecules moving through cytoplasm was observed to be as large as 10 µm^2^/s [10]; (ii) If *bcd* mRNA, and hence synthesis of Bcd, are localized at the anterior pole of embryo, Bcd has to diffuse as fast as 10 µm^2^/s to form a suitable Bcd gradient in a realistic time scale. The smaller value is used, on the other hand, for the intra-nuclear diffusion constant as *D*_nuc_<1.0 µm^2^/s. The use of a larger value of 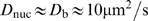 will result in 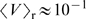, and the fluctuation of the *hb* expression will be largely reduced with such a large value of 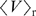. *D*_nuc_, however, is the diffusion constant in a short time scale in a much denser medium than cytoplasm, which should be nearer to the observed diffusion constant of 0.3–1.0 µm^2^/s in cortex [11],[12], so that the large value of 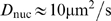 seems not appropriate and instead we should adopt the value 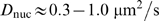, which leads to a small 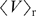 as used in Table 1.

**Table 1:** Standard Parameterization in the One-Dimensional Model.

Parameter		Standard value
*J*	Bcd synthesis rate	84.7 s^−1^
*D*_b_	Bcd diffusion constant	10 µm^2^ s^−1^
*k*_b_	Bcd degradation rate constant	7.7×10^−4^ s^−1^
<*h*_b_>	Bcd binding rate constant to the *hb* enhancer	0.19 s^−1^
*f*_b_	Bcd dissociation rate constant from the *hb* enhancer	7.7×10^−3^ s^−1^
*D*_h_	Hb diffusion constant	0.1 µm^2^ s^−1^
*k*_h_	Hb degradation rate constant	3.85×10^−4^ s^−1^
<*h*_h_>	Hb binding rate constant to the *hb* enhancer	5.8×10^−2^ s^−1^
*f*_h_	Hb dissociation rate constant from the *hb* enhancer	3.9×10^−3^ s^−1^
*g*(0)	Hb synthesis rate at *S* = 0	1.25×10^−6^ s^−1^
*g*(1)	Hb synthesis rate at *S* = 1	1.25×10^−6^ s^−1^
*g*(2)	Hb synthesis rate at *S* = 2	2.5×10^−2^ s^−1^
*g*(3)	Hb synthesis rate at *S* = 3	1.25 s^−1^
*N*_burst_	Burst size of Hb synthesis	10 molecules
*v*_ex_	Export rate constant of Hb or Bcd to interaction volume	0.4 s^−1^
<*v*_im_>	Import rate constant of Hb or Bcd from interaction volume	5.04×10^−4^ s^−1^
<Δ*x*>	The average mesh size	5 µm
<*V*_r_>	The average ratio of interaction volume to nuclear volume	10^−2.9^

After finishing preparation of the present manuscript, we became aware of the recent experimental report [41] suggesting that *bcd* mRNA is not strictly localized at the anterior pole but forms a gradient along the AP axis. With such a gradient of *bcd* mRNA, the smaller *D*_b_ might be enough to explain the observed Bcd gradient. Intense interest has been focused on the diffusion constant of Bcd [11],[14],[41], but consensus has not yet been achieved on either of the value of diffusion constant or the role of cytoplasmic diffusion of Bcd. We here adopt assumptions of the localized *bcd* mRNA and the large *D*_b_ as have been traditionally assumed in theoretical models [10], [13], [16]–[20],[36]. Further discussion on the value of diffusion constant will be given in *Discussion* section of the present paper.

We emphasize that embryo is not in a steady state but changes dynamically during development. We decompose problems, however, into two categories; dynamical problems and problems of accurate reading out. The dynamical problems will be considered in the more realistic three-dimensional model, and with the simplified one-dimensional model, we concentrate on the problems of accurate reading out by assuming the stationary nuclear configuration. This simplification in the one-dimensional model allows the extensive survey of the input/output relation over the wide parameter range. With the one-dimensional model, stochastic simulations were performed to reach the steady state and the data obtained at the last step of simulation were used for analyses. Then, the simulation was repeated 100 times to take the ensemble average. Dynamical quantities will be examined in the three-dimensional model introduced later in this paper, which has the appropriate time scale and is suited to examine the temporal change of quantities. Results of the one-dimensional model will be checked by the three-dimensional model to confirm whether the decomposition of problems into dynamic and static ones correctly describes the important features of embryos.

### Self-averaging of output: resolution of the paradox of signal interpretation

**Figure 3:**
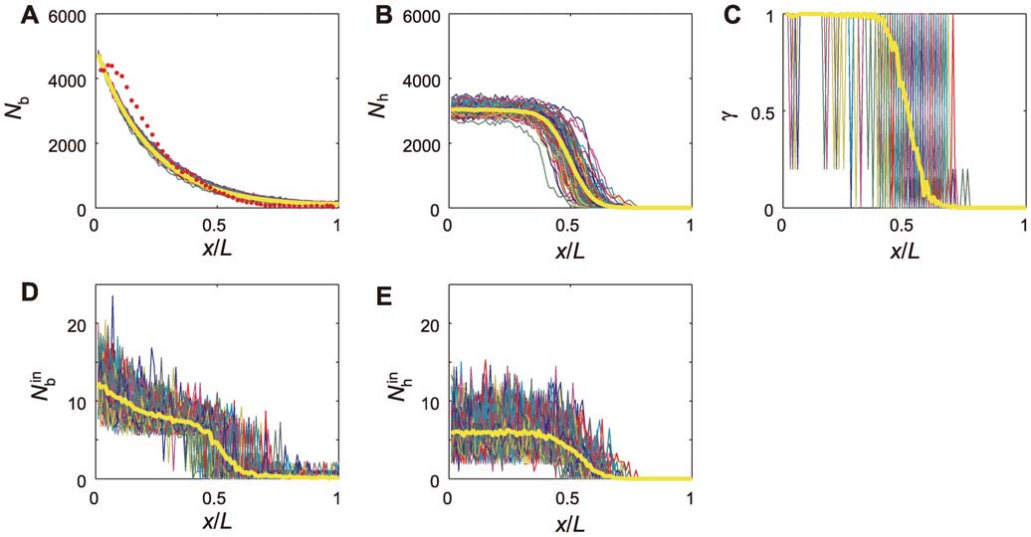
Profiles of Bcd, Hb, and the *hb* state in the one-dimensional model. All data (A–E) are plotted along positions 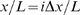 on the AP axis, and all statistics are calculated from the ensemble of 100 simulation runs. (A) Bcd profiles in the *i*th site, 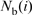, for 100 simulation runs are superposed and their average 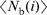 is shown with the yellow line. The observed data of the number of Bcd molecules in nuclei are plotted with red dots, which are averages taken over the ensemble of 15 embryos (Figure 5A of Ref.12). (B) Hb profiles in the *i*th site, 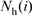, for 100 simulation runs are superposed and their average 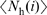 is shown with the yellow line. (C) Profiles of the *hb* enhencer state, 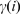, for 100 simulation runs are superposed and their average 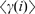 is shown with the yellow line. (D) Profiles of Bcd inside the interaction volume in the *i*th site, 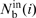, for 100 simulation runs are superposed and their average 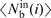 is shown with the yellow line. (E) Profiles of Hb inside the interaction volume in the *i*th site, 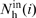, for 100 simulation runs are superposed and their average 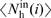 is shown with the yellow line.

In Figure 3A 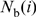 calculated with the one-dimensional model with the standard parameterization is plotted as a function of position, 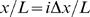. Here, results of 100 simulation runs are superposed and their average 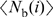 is compared with the observed data (Figure 5A of Ref.12). The panel shows that adjustment of a parameter *J* enables the model to consistently reproduce the observed Bcd profile in a semi-quantitative way. Plotted in Figure 3B is 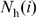 for 100 simulation runs and their average 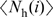. Plotted in Figure 3C is 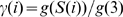, which measures the relative gene activity at the *i*th site, and its average 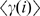. 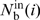 and 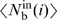 are plotted in Figure 3D and 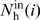 and 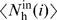 are plotted in Figure 3E. We see that fluctuations in 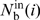 and 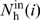 are much more significant than in 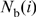 and 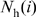 as expected from the smallness of 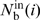 and 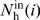. These large fluctuations are responsible for the large fluctuation of 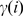 shown in Figure 3C. In spite of such large fluctuation in 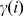, fluctuation in 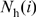 seems to be moderate.

**Figure 4:**
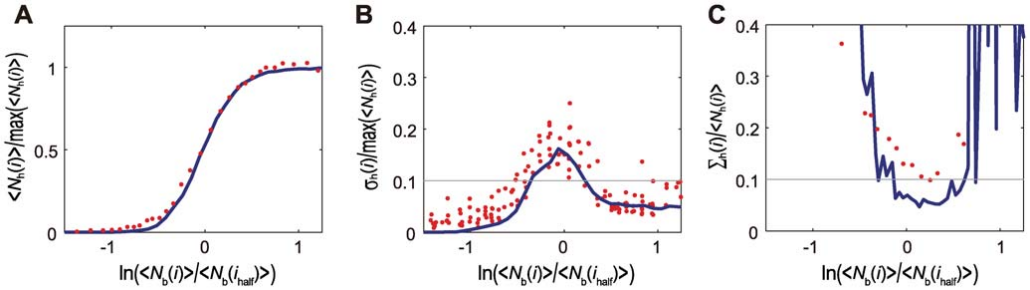
Input/output relation and its noise in the one-dimensional model with the standard parameterization. (A) The mean output 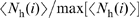 is plotted as a function of logarithm of the mean input, 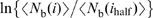 (blue line), where 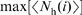 is the maximal value of 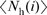 and *i*_half_ is defined by 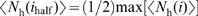. Red dots are the experimental data of the mean input/output relation averaged over nine embryos (Figure 4A of Ref.12). (B) Standard deviation of Hb levels for 100 simulation runs, 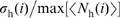, is plotted as a function of the input Bcd level (blue line). Red dots are the experimental data (Figure 4B of Ref.12). (C) Translation of the output noise of (B) into an equivalent input noise 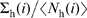 (blue line). Red dots are the experimental data (Figure 4C of Ref.12).

Fluctuation in 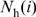 can be quantitatively analyzed with the input/output (IO) relation by regarding Bcd as input and Hb as output. We define the mean input as 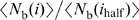, where *i*_half_ is the position satisfying 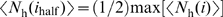 and 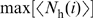 is the maximal value of 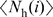 in the system. The mean output is defined by 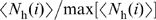. Thus defined mean IO relation is plotted in Figure 4A, which agrees well with the experimental data (Figure 4A of Ref.12). Fluctuation in the IO relation is shown in Figure 4B, where we plot 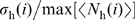 as a function of 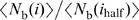 with 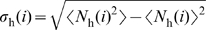. We find that the amplitude of fluctuation shown in Figure 4B is about 10%, which is as small as was observed in experiment (Figure 4B in Ref.12). The fluctuation of output data shown in Figure 4B can be converted to the form of equivalent input data as in Figure 4C by using the relation 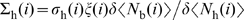, where 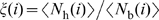, 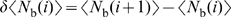 and 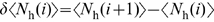. We can compare 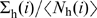 with 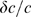 of Equation 1 to see whether the output fluctuation is as large as that expected in Bialek's argument: As plotted in Figure 4C, the calculated fluctuation in output, 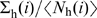, is around 10%, which is as small as was observed in experiment (Figure 4C of Ref.12) and is much smaller than the value expected in the argument of Equation 1. Thus, the present one-dimensional model shows that either the random diffusion of Bcd and Hb or the noisy expression of *hb* does not bring about large fluctuations in the IO relation.

It should be noted that in the one-dimensional model the IO relation is calculated by averaging quantities over the ensemble which is different from that in the experimental data: In experiment, many stripes along the AP axis of a single embryo can be used as an ensemble of data to measure the fluctuation of Bcd or Hb concentration in single embryo, and the small IO fluctuation observed in this ensemble is denoted by “preciseness” in Ref.12. In the present one-dimensional model, many runs of simulation are necessary to obtain the ensemble of enough size, and we here regard the smallness of fluctuations in this ensemble of many simulation runs as “preciseness” in the readout process. The same ensemble as in the experimental measurement can be taken in the three-dimensional model, and we will show later that the results of the three-dimensional model are essentially same as those of the present one-dimensional model, which should validate the use of this ensemble in the one-dimensional model to analyze precision in the readout process.

**Figure 5:**
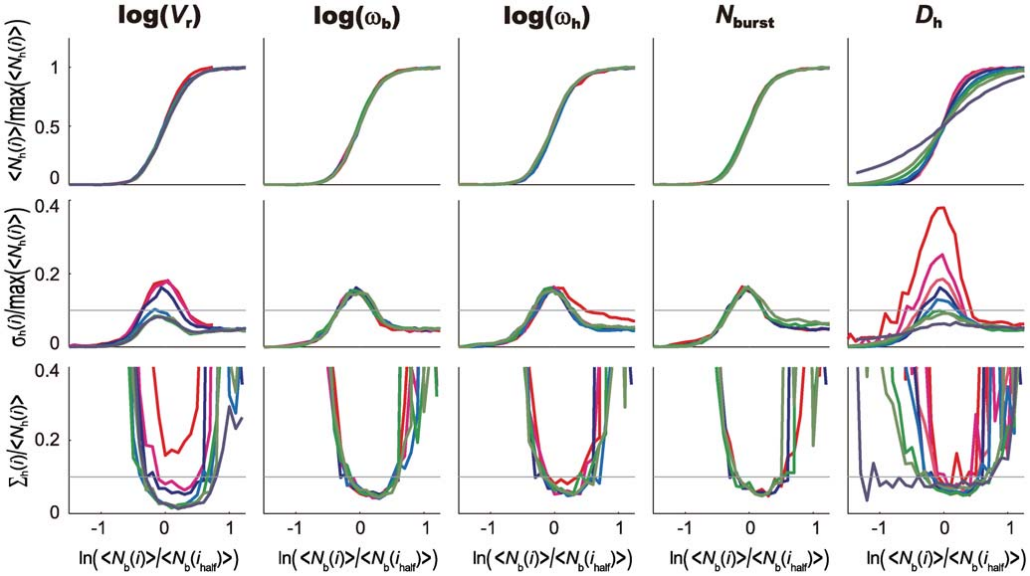
Input/output relations and their noise in the one-dimensional model with varied parameters. The same plot as in Figure 4 but with different parameterizations. For each simulation, one parameter is chosen from Table 1 to be varied from its standard value. (Left) The relative size of the interaction volume is varied as *V*_r_ = 10^−1^ (navy), 10^−1.5^ (dark green), 10^−2^ (light green), 10^−2.5^ (light blue), 10^−2.9^ (blue, standard value), 10^−3^ (magenta), and 10^−3.5^ (red). (Second Left) The dissociation rate of Bcd from the *hb* enhancer is varied as *ω*_b_ = *f*_b_/*k*_b_ = 10^4^ (dark green), 10^3^ (light green), 10^2^ (light blue), 10 (blue, standard value), 1 (magenta), and 10^−1^ (red). (Middle) The dissociation rate of Hb from the *hb* enhancer is varied as *ω*_h_ = *f*_h_/*k*_h_ = 10^4^ (dark green), 10^3^ (light green), 10^2^ (light blue), 10 (blue, standard value), 1 (magenta), and 10^−1^ (red). (Second Right) The number of Hb molecules synthesized in a burst is varied as *N*_burst_ = 100 (dark green), 50 (light green), 10 (blue, standard value), and 1 (red). The frequency of bursting, *g*(*S*), is also varied to keep *g*(*S*)*N*_burst_ constant. (Right) The diffusion constant of Hb is varied as *D*_h_ = 5 µm^2^/s (navy), 1 µm^2^/s (dark green), 0.5 µm^2^/s (light green), 0.25 µm^2^/s (light blue), 0.1 µm^2^/s (blue, standard value), 0.05 µm^2^/s (orange), 0.01 µm^2^/s (magenta), and 0 (red).

Effects of the parameter variation on the precision can be examined by changing parameters one by one from values in the standard parameterization. In Figure 5, the IO relation is analyzed by changing parameters, *V*_r_, *f*_b_, *f*_h_, *N*_burst_, and *D*_h_. Parameters *f*_b_ and *f*_h_ define the rate of change in the *hb* enhancer status. Instead of using the bare values of *f*_b_ and *f*_h_, we here use the normalized forms of *ω*_b_ = *f*_b_/*k*_b_ and *ω*_h_ = *f*_h_/*k*_h_ to explain the results. From computational [25],[28] and theoretical [24] investigations, we can expect that decrease in the value of *ω*_b_ or *ω*_h_ leads to increase in noise in the Hb synthesis. The large *N*_burst_ can also be a source of noise in the Hb synthesis [22]. Results of Figure 5 indicate, however, that changes in these parameters do not strongly affect the IO relation unless too small *ω*_b_ or *ω*_h_ is used. When *V*_r_ or *D*_h_ is varied, on the other hand, the clear change of the IO relation can be found: Smaller *V*_r_ gives the larger noise in the IO relation, showing that the random diffusion of Bcd and Hb are dominant sources to limit the precision of readout as was suggested by Gregor et al. [12]. *D*_h_ = 0 corresponds to the case that the spatial heterogeneity of *hb* expression is directly reflected in the Hb distribution. In this case the fluctuation in the IO relation is large as was expected from the argument of Gregor et al. [12]: The random diffusion and random reception at the *hb* enhancer lead to the fluctuation larger than the observed data, which exceeds the required precision to distinguish neighboring nuclei.

**Figure 6:**
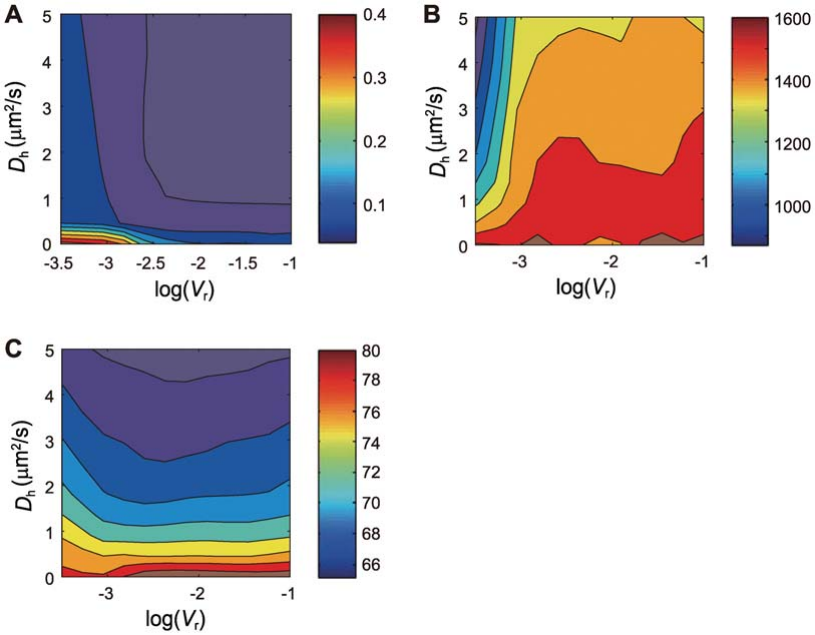
The input/output noise and the mean Hb profile in the one-dimensional model with varied parameters. The noise in the Input/Output relation and features of the mean Hb profile are plotted in the two dimensional plane of the Hb diffusion constant *D*_h_ and the relative size of the interaction volume *V*_r_. Other parameters are set to have standard values. (A) The maximal value of the standard deviation of Hb levels, 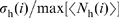 evaluated from the ensemble of 100 simulation runs, is plotted in the contour map. (B) 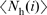 at the threshold position *x* = *x*_1/2_ = *i*_half_Δ*x*. (C) Angle *θ* of the Hb profile at the threshold position *x* = *x*_1/2_. Here, *θ* is calculated as 
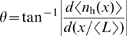
, and 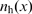 is a function of *x* = *i*Δ*x* obtained by fitting it to the calculated data of 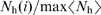.

These dependences on parameters are further examined in Figure 6. In Figure 6A, sensitive dependence of the fluctuation of Hb concentration on *V*_r_ and *D*_h_ is shown. When we assume the non-diffusive Hb with *D*_h_ = 0, the calculated results show the small enough amplitude of fluctuation only when *V*_r_ is larger than 10^−2^. With the realistic value of 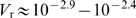, small but finite *D*_h_ with *D*_h_ 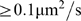 is necessary to explain the observed 10–20% fluctuation in the readout process. Effects of changing *ω*_b_ and *ω*_h_ were also examined (Figure S1): Small enough values of *ω*_b_ and *ω*_h_ bring about the large fluctuation, but in the parameter region of *ω*_b_>1 and *ω*_h_>1, the fluctuation amplitude is moderate and does not much depend on *ω*_b_ or *ω*_h_. In Figures 6B and 6C, dependence of the profile of Hb distribution on *V*_r_ and *D*_h_ is examined. The number of Hb molecules become very small when *V*_r_≪10^−3^ and *D*_h_>0.5 µm^2^/s, but does not sensitively depend on *V*_r_ or *D*_h_ when *V*_r_>10^−3^ or *D*_h_<0.5 µm^2^/s (Figure 6B). The angle of gradient of Hb distribution, on the other hand, sensitively depends on *D*_h_ (Figure 6C). From this dependence of the angle on *D*_h_, we see that *D*_h_ should be less than 0.3 µm^2^/s to explain the observed experimental profile of Hb distribution [12]. Diffusion constant of *D*_h_<0.3 µm^2^/s is consistent with the data that Hb does not diffuse over the long distance during a nuclear cycle [36].

**Figure 7:**
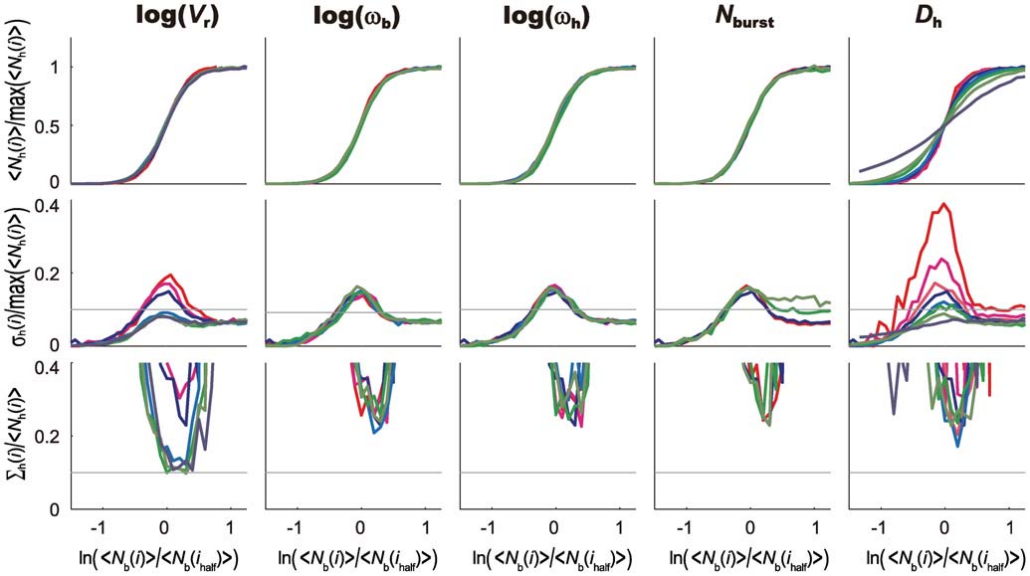
Input/output relations and their noise in the one-dimensional model with varied parameters for the case of no feedback regulation of *hb*. The same plot as in Figure 5 but Hb is assumed not to bind to the *hb* enhancer with *h*_h_ = 0.

Sensitive dependence of the fluctuation of Hb distribution on *V*_r_ is the result expected from the argument of Gregor et al. [12] and Bialek et al. [30]–[32], but the suppression of fluctuation by nonzero *D*_h_ is rather unexpected. To understand the reason why the small but finite *D*_h_ suppresses the Hb fluctuation, we performed simulation by using Equation 8, the assumption of disability of Hb to bind to DNA. With this assumption, the positive feedback loop of the *hb* regulation is lost and the cooperativity at binding sites of the *hb* enhancer is reduced. By putting *h*_h_ = 0 and prohibiting the state *S* = 3, the number of produced Hb decreases to about 25% of the amount calculated with the standard parameterization as was observed in the experiment of Ref.40. The slope angle of the Hb concentration at the threshold position becomes slightly smaller due to the decrease of cooperativity in the *hb* activation, which is consistent with the observation of Ref.36. The features of fluctuation in the IO relation, however, do not show a significant difference from the results obtained with the standard parameterization. In Figure 7, sensitive dependence of fluctuation in the IO relation on *V*_r_ and *D*_h_ in the case of *h*_h_ = 0 is shown. The fluctuation is dominated by the small *V*_r_ effect, but is substantially suppressed by the small but finite diffusion constant of Hb with 0<*D*_h_<0.3 µm^2^/s. Thus, we conclude that not the feedback loop of the *hb* regulation but the self-averaging due to the diffusion of Hb is sufficient to suppress the large fluctuations in the *hb* expression. With *D*_h_≈0.1 µm^2^/s, Hb molecules synthesized at around each nucleus are averaged over positions of several neighbor nuclei through diffusion. This simple mechanism is sufficient to suppress the fluctuation in the Hb distribution and hence resolves the paradox of signal interpretation.

### Three-dimensional model of *Drosophila* embryo

#### The three-dimensional system

**Figure 8:**
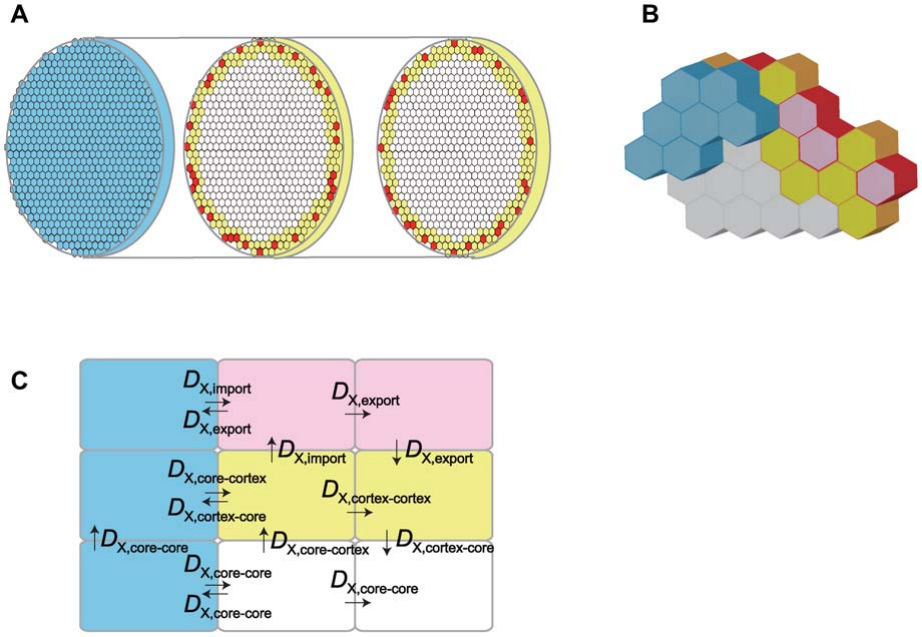
Three-dimensional model of *Drosophila* embryo. (A) An embryo is modeled by a cylinder of radius *R*≈90 µm and length *L*≈490 µm extending along the anterior-posterior axis. The cylinder is composed of 70 plates, each of which has thickness of Δ*x* = *L*/70 and consists of 625 hexagonal sites: The system consists of 70×625 = 43750 sites in total. These sites are classified into three types; nuclear sites (red), cortical sites (yellow), and core sites (white). Sites in the most anterior slice are core sites, from which Bcd is synthesized (blue). (B) Close-up of a part of the most anterior sites and sites in the next slice in the model. (C) Diffusion constants of molecule X can have different values *D*_X, Y-Z_, depending on the type of starting site Y, and the type of arriving site Z. Types of sites are represented by the same colors as in A and B. We write X = b for Bcd and X = h for Hb molecules. *D*_X, core-nuclear_ is denoted by *D*_X, import_ and *D*_X, nuclear-core_ is denoted by *D*_X, export_. Values of these constants are listed in Table 2.

Effects of the dynamically changing nuclei are examined by developing the three-dimensional model. As shown in Figures 8A and 8B, an embryo is represented by a cylinder of length *L* extending along the AP axis. 15 embryos were sampled in Refs.11 and 12, and their average size was found to be about 490 µm. In these 15 embryos, about 70 nuclei were distributed at the intersection of the midsagittal plane. In order to quantitatively compare the simulated results with these observed data, we use the system having the similar size: We consider the cylinder composed of 70 plates each of which has the thickness of Δ*x* = *L*/70 with *L*≈490 µm. Each plate has an approximately circular shape of radius 90 µm, and consists of 625 hexagonal sites whose side length is *β*≈4 µm. The system, therefore, consists of 70×625 = 43750 sites. Starting from *t* = 0 at the time of oviposition, the dynamical change of distribution of Bcd and Hb is followed with the stochastic simulation until *t* = 144 min at the interphase of nuclear cycle 14. This simulation was repeated 10 times by varying *β* and Δ*x* around averages 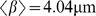 and 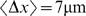 with deviations of 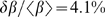 and 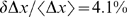 to simulate the observed fluctuation of 4.1% in size of embryo [12]. The system has three types of sites; cortical, core, and nuclear sites. Each nuclear site contains a single nucleus and other types of sites do not contain nucleus. Position of the site is designated by (*i*, *j*), where *i* is the number of slice from *i* = 1 at the anterior slice to *i* = 70 at the posterior slice. *j* = 1–625 designates position of the site in the *i*th slice. The numbers of Bcd and Hb in each site at time *t* are denoted by 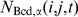 and 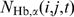, where *α* = cortical, core, or nuclear specifies the type of site. Diffusion and reactions defined at sites depend on types of sites.

We should keep in mind that the shape of embryo is more like a prolate ellipsoid and that cylinder is just a 0th order approximation to it. Due to this disagreement in shape of the system, the simulated Bcd and Hb distributions at the anterior pole should deviate from the observed distributions. These deviations, however, should be localized at the anterior pole and are not important to examine the global features of the Bcd gradient and the boundary of the Hb distribution observed at around the middle of embryo. The present cylindrical model, therefore, provides a computationally efficient tool to study the global Bcd and Hb distributions in embryo. See Text S1 for more detailed explanation of the model.

#### Diffusion and reactions

Diffusion and reactions of Bcd and Hb defined in the three-dimensional model are the straightforward extension of those considered in the one-dimensional model. Bcd and Hb diffuse between adjacent lattice sites in the three-dimensional space as in the same way as in the one-dimensional model. Diffusion constants, however, depend on types of starting or arriving sites, which are explained in more details in Figure 8C and in *Method* section. Bcd and Hb are degraded with the rate of 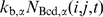 and 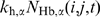. Here, 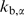 and 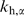 are assumed to be small in cortical or core sites but large in nuclear sites. At the core sites in the slice *i* = 1, Bcd is generated with the rate *J*(*m*) with *m* representing the nuclear cycle. Since the observed production rate of Bcd increases as nuclear cycles proceed [42], we assume *J*(*m*) to be an increasing function of *m*.

As in the one-dimensional model, effects of random reception of Bcd or Hb molecules at the *hb* enhancer are simulated by introducing the nanometer scale spatial region of interaction volume. We use *V*_r_ to designate the ratio of interaction volume to the volume of a nucleus, so that the smallness of *V*_r_ is the source of fluctuations in the *hb* expression. 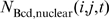 and 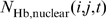 are decomposed into three parts as in the one-dimensional model depending on whether the molecule is in or out of the interaction volume, and on whether the molecule is bound to the *hb* enhancer or not, so that
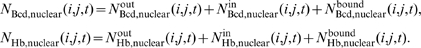
(9)The *hb* enhancer state in the three-dimensional model is *S*(*i*, *j*, *t*) = 0–3. Hb is synthesized at around nuclei, i.e. in nuclear sites with the rate *g*(*S*(*i*, *j*, *t*)). Values of *g*(*S*(*i*, *j*, *t*)) can be determined in the same way as in Equation 7 or Equation 8. Further details on parameters are explained in *Method* section and are summarized in Table 2. We refer to the parameterization of Table 2 as the standard parameterization in the three-dimensional model.

**Table 2:** Standard Parameterization in the Three-Dimensional Model.

Parameter		Standard value
*J*	Bcd synthesis rate	*J*(*m*) s^−1^
*D*_b,import_	Bcd diffusion constant (import into nucleus)	1.2 µm^2^ s^−1^
*D*_b.export_	Bcd diffusion constant (export from nucleus)	0.3 µm^2^ s^−1^
*D*_b,core-cotex_	Bcd diffusion constant (from core to cortex)	12.5 µm^2^ s^−1^
*D*_b,cortex-core_	Bcd diffusion constant (from cortex to core)	12.5×*Q*(*m*) µm^2^ s^−1^
*D*_b,cortex-cortex_	Bcd diffusion constant (from cortex to cortex)	12.5 µm^2^ s^−1^
*D*_b,core-core_	Bcd diffusion constant (from core to core)	18.0 µm^2^ s^−1^
*k*_b, nuclear_	Bcd degradation rate constant in nuclei	7.70×10^−4^ s^−1^
*k*_b, cortical_, *k*_b, core_	Bcd degradation rate constant in cytoplasm	9.63×10^−5^ s^−1^
<*h*_b_>	The average Bcd binding rate constant to the *hb* enhancer	7.70×10^−3^ s^−1^
*f*_b_	Bcd dissociation rate constant from the *hb* enhancer	7.70×10^−2^ s^−1^
*D*_h,import_	Hb diffusion coefficient (import into nucleus)	0.1 µm^2^ s^−1^
*D*_h,export_	Hb diffusion coefficient (export from nucleus)	0.1 µm^2^ s^−1^
*D*_h,core-cortex_	Hb diffusion coefficient (from core to cortex)	0.1 µm^2^ s^−1^
*D*_h,cortex-core_	Hb diffusion coefficient (from cortex to core)	0.1 µm^2^ s^−1^
*D*_h,cortex-cortex_	Hb diffusion coefficient (from cortex to cortex)	0.1 µm^2^ s^−1^
*D*_h,core-core_	Hb diffusion coefficient (from core to core)	0.1 µm^2^ s^−1^
*k*_h, nuclear_	Hb degradation rate constant in nuclei	3.85×10^−4^ s^−1^
*k*_h, cortical_, *k*_h, core_	Hb degradation rate constant in cytoplasm	4.81×10^−5^ s^−1^
<*h*_h_>	The average Hb binding rate constant to the *hb* enhancer	1.85×10^−2^ s^−1^
*f*_h_	Hb dissociation rate constant from the *hb* enhancer	3.85×10^−2^ s^−1^
*g*(0)	Hb synthesis rate at *S* = 0	7.50×10^−6^ s^−1^
*g*(1)	Hb synthesis rate at *S* = 1	7.50×10^−6^ s^−1^
*g*(2)	Hb synthesis rate at *S* = 2	1.50×10^−1^ s^−1^
*g*(3)	Hb synthesis rate at *S* = 3	7.50×10^−1^ s^−1^
*N*_burst_	Burst size of Hb synthesis	10 molecules
*v*_ex_	Export rate constant of Hb or Bcd from interaction volume	2.04×10^−2^ s^−1^
<*v*_im_>	The average import rate constant of Hb or Bcd to interaction volume	5.98×10^−5^ s^−1^
<*V*_r_>	The average ratio of interaction volume to nuclear volume	10^−2.5^
<*β*>	The average side length of hexagon of each site	4.04 µm
<*Δx*>	The average side length of the site along the AP axis	7 µm

The factor *J*(*m*) at the nuclear cycle *m* is *J*(*m*) = 0.1×exp(ln(100)/14×10) for *m* = 1–10 and 0.1×exp(*m* ln(100)/14) for *m* = 11–14. The factor *Q*(*m*) at the nuclear cycle *m* is *Q*(*m*) = 1 (for *m* = 1–8), 0.667 (for *m* = 9), 0.444 (for *m* = 10), 0.198 (for *m* = 11), 0.132 (for *m* = 12), and 0.088 for (*m* = 13–14).

#### Rules of mitoses and growth of cortical layer

Nuclei undergo nuclear cycles, increase in number, and change their locations in embryo. One nuclear cycle consists of the interphase period and the mitotic period. During the interphase period, nucleus is stable, which is described by a nuclear site in the model. During the mitotic period, nuclear membrane is dissolved and nuclear sites are turned into cortical or core sites in the model. After the mitosis, nuclear membrane is rebuilt to enter into the next nuclear cycle, and the number of nuclear sites is doubled. Since it was observed that somatic nuclei in *Drosophila* embryo undergo nearly synchronous nuclear divisions as a syncytium [43], we treat these nuclear cycles as synchronized events in the model.

At *t* = 0, one nuclear site is defined at around the center of embryo and nuclear cycle 1 is initiated. During nuclear cycle *n* = 2 to 7, the number of nuclei increases as 2*^n^* ^−1^. The increased nuclei are placed randomly at around the center of embryo. At the 8th nuclear cycle, about 90 nuclei of total 2^7^ = 128 nuclei begin to move to the surface region of embryo and the remaining nuclei are left in the middle of embryo [43],[44]. In the model, about 90 nuclear sites are redistributed randomly in the region somewhat apart from the center of the cylinder. The nuclei left in the center are yolk nuclei [43],[44], which are turned into the core sites in the model. About 90 nuclei departing from the center reach the surface region at nuclear cycle 10 and are doubled in number through nuclear cycle 14. With this rule of mitoses and movement, the surface of the cylinder in the model is occupied by about 5500–6200 nuclear sites at the interphase period of nuclear cycle 14 depending on the number of nuclei selected to move toward the cylinder surface at nuclear cycle 8. More detailed explanation of rules is given in Text S1. During the interphase period, volume of nuclei increases in *Drosophila* embryo [11] but here we neglect effects of the nuclear volume change and keep the size of nuclear site constant for the sake of simplicity.

Sites near the cylinder surface are defined as cortical sites if they are not nuclear sites. The cortical sites form a single layer of sites on the surface of cylinder until nuclear cycle 10, and the thickness of cortical layer increases as nuclear cycle proceeds from 11 to 14. See Text S1 for the further explanation on cortical sites. Sites other than cortical or nuclear sites are core sites. In this way the dynamical change in embryonic structure is represented by rules defined at each time step in the model. We perform stochastic simulations of diffusion and reactions of Bcd and Hb under these rules to see how such dynamically changing embryonic environment affects the stability, precision and reproducibility of distributions of Bcd and Hb.

#### Simulation with the three-dimensional model

With the three-dimensional model, stochastic simulations of diffusion and reactions are performed. 10 simulation runs were repeated by allowing the fluctuation of embryonic size with 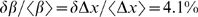. In order to compare the calculated results with the observed data, quantities are normalized by the factor 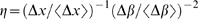 as 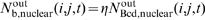, 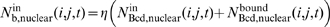, 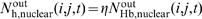, 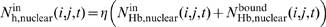, 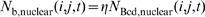, and 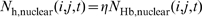.

### Reproducible patterning in multiple embryos

**Figure 9:**
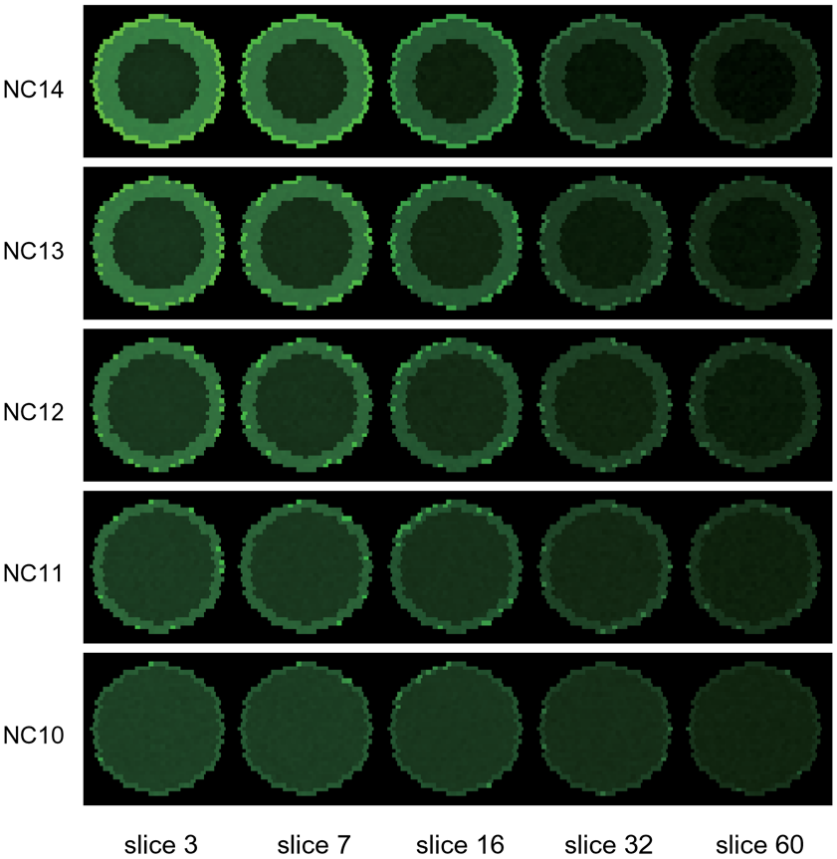
Snapshots of the Bcd distribution in the three-dimensional model. Concentration of Bcd in each site is expressed by green shaded color. Slices at *i* = 3, 7, 16, 32, and 60 are shown at nuclear cycle 10–14.

Shown in Figure 9 are snapshots of a simulation run with the three-dimensional model, in which the Bcd concentration is represented by green shaded colors. As the mitoses are repeated from nuclear cycle 10 to 14, the number of nuclei on the surface of embryo increases and the cortical layer becomes thick. Bcd is concentrated in nuclei which can be recognized as the bright green dots and is also accumulated in the cortical layer to exhibit the green shaded region. These features are similar to the observed snapshots in distribution of Bcd-eGFP (Figure 6A of Ref.11).

**Figure 10:**
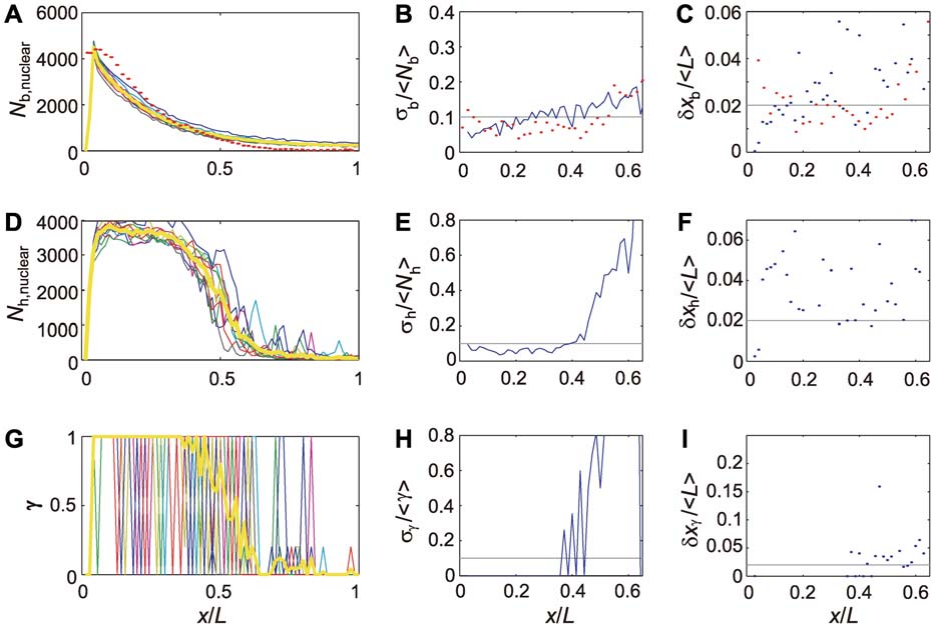
Reproducibility of profiles of Bcd, Hb, and the *hb* state in the three-dimensional model. All data are plotted along positions 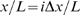 on the AP axis. In simulation, all statistics are calculated from the ensemble of 10 simulation runs. The compared experimental statistics of Ref.12 were obtained from the ensemble of 15 embryos. (A) Bcd profiles 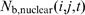 in a stripe of the *j* = 100th sites in slices *i* = 1–70 at time *t* = 144 min. Data of 10 simulation runs are superposed. The *j* = 100th sites are on the surface of the cylinder. The average of these 10 curves, 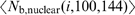, is drawn with the yellow line. The observed data of averaged Bcd numbers in nuclei (Figure 5A of Ref.12) is plotted with red dots. (B) The standard deviation of 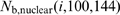 is plotted in the normalized form as 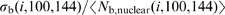 (blue line). The observed standard deviation (Figure 5B of Ref.12) is plotted with red dots. (C) Variability of Bcd profiles is translated into an effective positional fluctuation 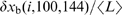 (blue dots). The observed positional fluctuation (Figure 5C of Ref.12) is plotted with red dots. (D) Hb profiles 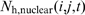 in a stripe of the *j* = 100th site in slices *i* = 1–70 at time *t* = 144 min for 10 simulation runs are superposed. The average of these 10 curves, 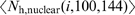, is drawn with the yellow line. (E) The standard deviation of 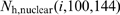 is plotted in the normalized form as 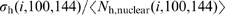. (F) Variability of Hb profiles is translated into an effective positional fluctuation 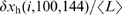. (G) Profiles of *hb* gene state, 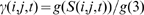 in a stripe of the *j* = 100th site in slices *i* = 1–70 at time *t* = 144 min for 10 simulation runs are superposed. The average of these 10 curves, 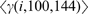, is drawn with the yellow line. (H) The standard deviation of 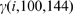 is plotted in the normalized form as 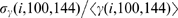. (I) Variability of profiles of *hb* state is translated into an effective positional fluctuation 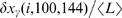. Horizontal lines in middle and right panels are drawn for guides of eyes.

 Profiles of distribution of Bcd and Hb and the distribution of the *hb* enhancer state are examined in Figure 10. In the left panels of Figure 10 (Figures 10A, 10D, 10G), results of 10 simulated embryos are superposed, each of which is obtained by monitoring nuclear sites along a stripe of the cylinder surface at an instance in the interphase period of nuclear cycle 14. In the middle panels of Figure 10 (Figures 10B, 10E, 10H), the embryo-to-embryo fluctuations of the Bcd distribution, 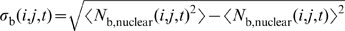, the Hb distribution, 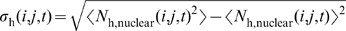, and the *hb* enhancer state, 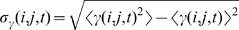 are plotted, where 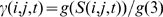 and 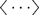 is the ensemble average over 10 embryos. We can see from Figure 10 that the embryo-to-embryo fluctuation of Bcd distribution is as small as 10% as in the observed data (Figure 5B of Ref.12). 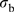, 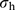, and 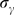 can be transformed to the positional fluctuations as 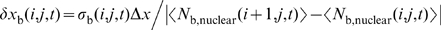, 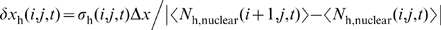, and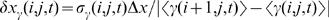, which are plotted in the right panels of Figure 10 (Figures 10C, 10F, 10I). Positional fluctuation of Bcd, 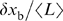, is comparable with the observed data (Figure 5C of Ref.12) showing that the Bcd gradient is generated in a reproducible way with small embryo-to-embryo fluctuation at the interphase of nuclear cycle 14. Fluctuations of the Hb distribution (Figure 10E) and the *hb* enhancer state (Figure 10H) are larger than fluctuation of Bcd (Figure 10B); At the threshold position of *x/L*∼0.48, 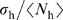 and 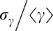 amount to 40–50%. The positional fluctuations, 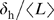 and 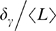, however, are less than 5% (Figures 10F and 10I) because of the sharp change in 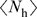 and 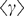 at the threshold position. Thus, Bcd, Hb, and the *hb* enhancer state are reproducible with the small embryo-to-embryo fluctuation.

### Mechanism of precise readout

**Figure 11:**
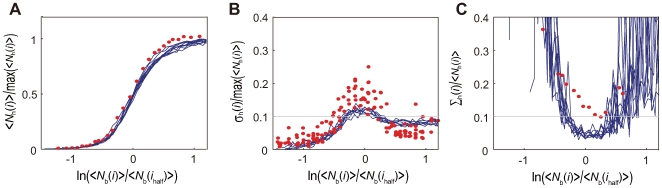
Input/output relations and their noise in the three-dimensional model with the standard parameterization. (A) Mean input/output relations for 10 simulation runs are superposed (blue lines). A mean input/output relation is obtained by plotting 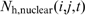 in one simulation as a function of 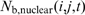 for all nuclei (about 6000 nuclei) in the system at *t* = 144 min and by binning them into 50 bins. Red dots are the experimental data (Figure 4A of Ref.12). (B) For each bin from (A) the standard deviation of 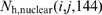 is calculated. Drawn are 10 lines of the standard deviation for 10 simulation runs. Red dots are experimental data of standard deviation of input/output relations for nine embryos (Figure 4B of Ref.12). (C) Translation of 10 lines of the output noise of (B) into lines of equivalent input noise 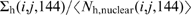 (blue lines). Red dots are the experimental data (Figure 4C of Ref.12).

We calculated the IO relation by taking the average over many nuclei in a single embryo at the interphase of nuclear cycle 14. Lines of the IO relation obtained from each of 10 runs of simulation are superposed in Figure 11A. These 10 lines do not deviate much from each other, which is the feature consistent with the experimental data (Figure 4A of Ref.12). Fluctuations in the IO relation are quantified in Figures 11B and 11C, which show a good agreement with the observed data (Figures 4B and 4C of Ref.12). Small values of each IO fluctuation shown by each line of Figures 11B and 11C imply that the Bcd gradient is read in a precise way in each embryo, and the small deviation of multiple IO lines from each other implies that the Bcd gradient is read out in a reproducible way with the small embryo-to-embryo fluctuation. In this way, results of the three-dimensional model show that both the generation of the Bcd gradient and the readout of the Bcd gradient are precise and reproducible at the nuclear cycle 14 in spite of the large dynamical structure change of embryo through nuclear cycle 1–14.

Dependence of the IO relation on parameters has also similar features to those discussed with the one-dimensional model (Figure S2). The fluctuation of the IO relation, *σ*_h_ at nuclear cycle 14, does not sensitively depend on *ω*_b_, *ω*_h_ or *N*_burst_ as far as *ω*_b_ or *ω*_h_ is not extremely small. The IO fluctuation is sensitive, on the other hand, to *V*_r_ and *D*_h_. Thus, the qualitatively same results as the one-dimensional model were obtained with the three-dimensional model, which supports the view that the fluctuation in the IO relation is dominated by the small value of *V*_r_, and the fluctuation is masked by the small but finite *D*_h_. When the positive feedback of Hb is turned off by posing *h*_h_ = 0, the amplitude of *N*_h_ decreases as in the one-dimensional model, but features of preciseness and reproducibility are not altered much (Figure S3), which implies that the positive feedback mechanism is not necessary to suppress the fluctuations.

This mechanism to resolve the paradox of signal interpretation can be visually confirmed by taking snapshots of the side-view of the model embryo (Figure S4). The *hb* enhancer state, *γ*, shows large spatial heterogeneity at the threshold region with about half of nuclei being randomly activated to produce Hb molecules for both two cases of *D*_h_ = 0.1 µm^2^/s (Figure S4A) and *D*_h_ = 0 (Figure S4B). When *D*_h_ = 0.1 µm^2^ s^−1^, thus produced Hb diffuses over several nuclei, which smoothes out this heterogeneity to produce a boundary of the Hb distribution (Figure S4C), which is in sharp contrary to the case of *D*_h_ = 0 for which the Hb distribution directly reflects the heterogeneity of the gene activity (Figure S4D).

Though *hb* mRNA is not explicitly taken into account in the present model, we may be able to expect that the noise level of the *hb* mRNA distribution is in between the noise level of distribution of the *hb* gene activity and that of the Hb distribution since mRNA first localizes in nuclei in which the *hb* gene is active but gradually diffuses out of nuclei to represent both characteristics of the localized *hb* gene activity and the more delocalized diffusive *hb* products. This expectation is consistent with the observed larger fluctuation of the *hb* mRNA distribution than the Hb distribution (Figure S8 of Ref.36).

### Stable patterning in dynamically changing embryo

**Figure 12:**
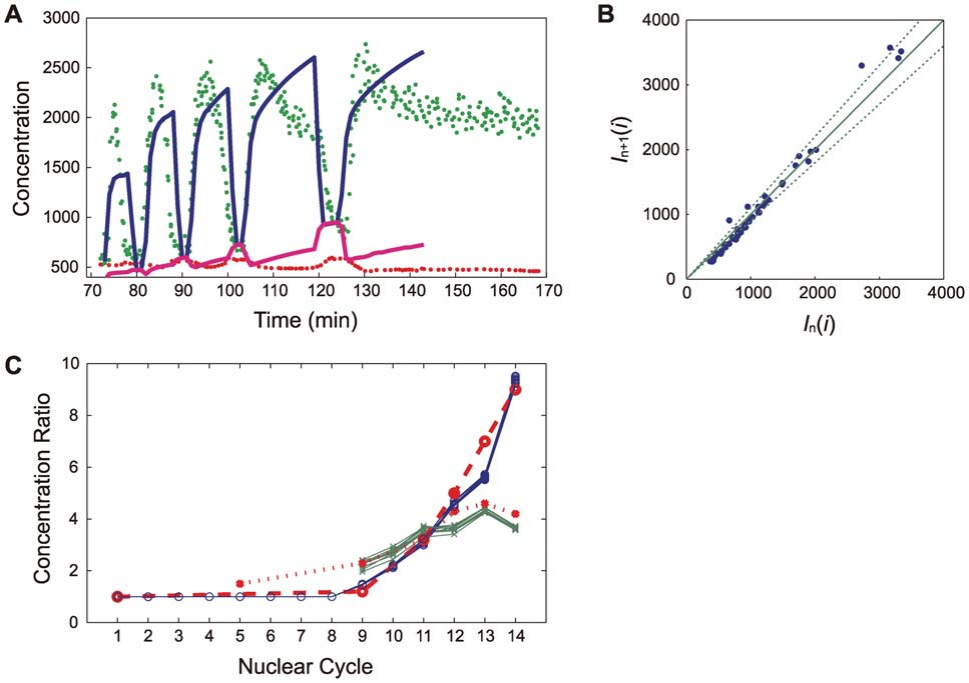
Temporal development of the Bcd distribution. (A) Nuclear and cytoplasmic development of the number of Bcd molecules from nuclear cycle 10 to 14. Numbers of Bcd molecules in nuclear sites at the *i* = 10th slice (*x*/*L* = 0.14), 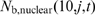, are averaged over *j* in the outmost layer of the cylinder and over 10 simulation runs (blue line), and numbers of Bcd molecules in cortical sites at the *i* = 10th slice (*x*/*L* = 0.14), 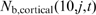, are averaged over *j* in the outmost layer of the cylinder and over 10 simulation runs (magenta line). Experimental data of Bcd concentrations (Figure 3D of Ref.11) at nuclei (blue dots) and at cortex (red dots) are compared. (B) Scatter plot of peak numbers of Bcd molecules in successive nuclear cycles. The maximum value of 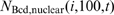 during nuclear cycle *n* is recorded as 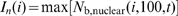 for slice *i* = 5, 10, 15…, 65, and then the recorded data are plotted at the coordinate (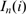, 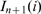) in the two-dimensional plane for *n* = 8–10. The *j* = 100th sites are on the surface of the cylinder. Line with the slope one corresponds to the perfect stability across nuclear cycles and dotted lines correspond to 10% deviation. (C) Development of ratio of concentrations. Numbers of Bcd molecules averaged over the whole system and over the time duration of interphase of nuclear cycle *n* are denoted by 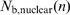, 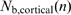, and 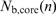, for nuclear, cortical and core sites, respectively. Plotted are 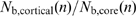 (blue line), and 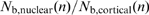 (green line). 10 lines for 10 simulation runs are superposed. The experimental data for (Bcd concentration in cortex)/(Bcd concentration in core) are plotted with red open circles and the experimental data for (Bcd concentration in nuclei)/(Bcd concentration in cortex) are plotted with red dots, both of which are taken from Figure 6D of Ref.11.

With the three-dimensional model, the simulated temporal change of the Bcd distribution during nuclear cycle 10–14 can be compared with the experimental data. In Figure 12A, the simulated Bcd concentration at the anterior part is compared with the experimental data [11]. Plotted are numbers of Bcd molecules in nuclear and cortical sites at the outmost layer of the cylinder in the slice *i* = 10 (*x*/*L* = 0.14). The Bcd concentration in nuclei is large during the interphase and sharply drops to the smaller values during mitoses. During the mitotic period, Bcd is released to the cortical layer and the Bcd concentration in cortical sites increases. In the observed data, the Bcd concentration in nuclei sharply rises in the beginning of the interphase period and then gradually decreases during the interphase period. Gregor et al. showed that this decrease in the Bcd concentration should be attributed to increase in size of each nucleus during the interphase period: With increase of the nuclear volume, Bcd is diluted in nuclei, which is partially offset by increase of incoming flux to the nuclei. Such dynamical balance among incoming/outgoing fluxes and dilution of Bcd should result in the gradual decrease of the Bcd concentration during the interphase period [11]. Since the growth of the nuclear size is not taken into account in the present model, decrease of Bcd concentration during the interphase period is not reproduced in simulation but the Bcd concentration gradually increases due to the active transport of Bcd into nuclei. Apart from such a difference, the overall features of the simulated results agree well with the observed data (Figure 3D of Ref.11). The Bcd concentration is stably reproduced in the next nuclear cycle even after Bcd is lost during the mitotic period. This stability is quantified by comparing the peak values of the Bcd concentration in successive nuclear cycles. In Figure 12B, the peak values in successive nuclear cycles are shown to differ only about 10%, showing that the Bcd concentration profile is as stable as was observed in experiment (Figure 3E of Ref.11). Such stable Bcd profile is realized because Bcd molecules released to cortical sites during the mitotic period are efficiently concentrated again into nuclei in the next nuclear cycle. The rapid transport of Bcd from cortex to nuclei is the necessary condition to prevent Bcd from escaping into core sites to achieve this efficient concentration. In Figure 12C, the simulated accumulation of Bcd in the cortical layer is compared with the experimental data (Figure 6D of Ref.11). The factor *Q*(*m*) defined in Table 2, which represents the gradual decrease of diffusion constant from cortex to core sites, was determined to reproduce this experimental plotting. See *Method* section for further explanation of *Q*(*m*). In Figure S5, the snapshots of the Bcd distribution are shown to visualize how Bcd is released in the mitotic period and is attracted into nuclei again in the interphase period.

The Hb distribution is also stable in dynamically changing embryo. By calculating the distribution of the number of Hb molecules along a strip on the cylinder for *t* = 126–143 min in the interphase period of nuclear cycle 14, we can see that the number of Hb molecules increases in a coherent manner along the AP axis as Bcd is being concentrated in nuclei and the Hb synthesis is promoted (Figure S6A). The distribution profile of Hb is established soon after the Hb synthesis starts, so that the threshold position *x*_1/2_/*L* = *i*_half_Δ*x*/*L* of the Hb distribution only slightly shifts during nuclear cycle 14 (Figure S6B), which is consistent with the small shift of *x*_1/2_/*L* in the experimental data (Figure 2C of Ref.36). The slope of the Hb distribution at *x*_1/2_ is kept steep during nuclear cycle 14 (Figure S6C), which roughly agrees with the experimental data (Figure 2D of Ref.36).

**Figure 13:**
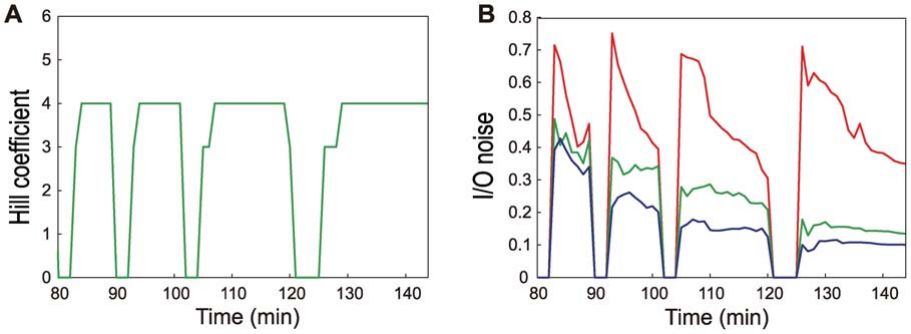
Development of the Hill coefficient and input/output noise. (A) Temporal change of the Hill coefficient *n* obtained by fitting the Hill relation to the input/output relation of Figure 11A. *n* is put to be 0 during mitoses. (B) Temporal change of the peak height of the average standard deviation of input/output relations. The standard deviation is obtained from ensemble of nuclei in the single embryo as lines in Figure 11B, and the average was taken for 10 data of the standard deviation of 10 simulation runs. The peak height is put to be 0 during mitoses. *D*_h_ = 0.5 µm^2^/s (blue), 0.1 µm^2^/s (green, standard value), and 0 (red).

The experimentally observed IO relation around *x*_1/2_ has been fitted by the Hill relation as
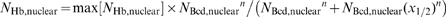
with the Hill coefficient *n* = 3–5 [12],[35],[36],[38]. Results of the same fitting of the simulated data are shown in Figure 13A during nuclear cycle 11–14. Through these nuclear cycles, *n* stays constant in interphase of different nuclear cycles in spite of the repeated mitoses between interphase periods. We should note that such stable Hb distribution should be realized in dynamically changing embryo only when the stable and precise patterning of Bcd distribution is established.

Temporal change of fluctuation of the IO relation in nuclear sites near the threshold position *x*_1/2_ is shown in Figure 13B: As shown in Figure 11B, the fluctuation of the IO relation shows a peak at *x*_1/2_. Plotted in Figure 13B is the time course of the peak height of the fluctuation of the IO relation. When *D*_h_ = 0, the fluctuation sharply increases at the onset of interphase in each nuclear cycle because multiplicated nuclei are placed at positions away from Hb molecules inherited from the previous nuclear cycle. As the busting production takes place multiple times, the Hb molecules are accumulated around nuclei and the Hb concentration approaches the steady level due to the balance between the Hb production and degradation, but the remaining fluctuation is still larger than the fluctuation observed in experiment [12]. When *D*_h_ is finite with *D*_h_ = 0.1–0.5 µm^2^/s, on the other hand, the fluctuation is much suppressed and reaches the experimentally observed level [12]. Here, the important feature is that the fluctuation for nonzero *D*_h_ decreases by taking a long time from 50–35% in nuclear cycle 11 to 15–10% in nuclear cycle 14. This long-time decrease is due to the slow diffusion of Hb. See also Video S1 to grasp the intuitive picture of how the slow diffusion of Hb averages the Hb distribution through multiple nuclear cycles to reach the homogeneous distribution in the surface layer of a slice of embryo. Though the larger *D*_h_ is more effective to reduce the fluctuation, too large *D*_h_ smears out the Hb distribution along the AP axis and flattens the slope of the profile at the boundary of *hb* activation as shown in Figure 6 for the one-dimensional model. To keep the sharpness of the Hb boundary, *D*_h_ has to be smaller than 0.3 µm^2^/s. As *D*_h_ is small, the self-averaging process of Hb should take time longer than the duration of a single nuclear cycle and multiple cycles are needed to reduce the fluctuation. In this way, the stable Bcd and Hb profiles lasting through multiple nuclear cycles is the basis to assure the effective reduction of fluctuation through multiple nuclear cycles.

## Discussion

One- and three-dimensional models of *Drosophila* embryo were developed and two major issues raised by the experimental visualization of embryonic development [11],[12] were examined with stochastic simulations: One of the issues is on the mechanism to generate the profile of Bcd gradient. We showed that the stochastic processes of synthesis, diffusion and degradation of Bcd give rise to the Bcd distribution whose entire profile remains stable through multiple nuclear cycles. This stable profile is precise to exhibit only the small fluctuation within each embryo and is reproducible with small fluctuation among multiple embryos. The stable profile of Bcd is realized by the rapid transport of Bcd to nuclei. The other issue is on the readout of the Bcd gradient. Random diffusion and reception of Bcd molecules at the nanometer scale region of DNA induce the intense fluctuation in the *hb* gene activity. Our models showed that this fluctuation indeed dominates fluctuation in the *hb* expression, but the fluctuation in the Hb distribution is masked by the slow diffusion of Hb molecules: The slow diffusion of Hb through multiple nuclear cycles averages the Hb distribution without losing the sharp boundary of distribution at around the threshold position. In this way, self-averaging due to the slow diffusion of the output protein resolves the paradox of signal interpretation and enables the precise and reproducible readout of the gradient. Since the self-averaging process of Hb has to continue over multiple nuclear cycles to achieve the sufficient accuracy, the stable Bcd distribution over multiple nuclear cycles is a necessary condition for realizing the accurate Hb distribution. Thus, the coordinated diffusion of input and output molecules is the basis to generate the stable, precise, and reproducible patterning of both input and output molecules.

Our prediction of the Hb self-averaging mechanism should be tested experimentally. Because mobility of the fused protein of Hb and the other protein domain should be affected by the characteristics of the added domain, diffusion constant of the Hb-fusion protein could be systematically changed by modulating the mass, size, and surface charge of the added domain. One possible example is to add a small tag such as histidine residues to eGFP, which can reduce velocity of the Hb-eGFP fusion protein [45]. Although such modulation may change the affinity of Hb to the *hb* enhancer to reduce effects of the feedback regulation of the *hb* expression, we should note that this feedback regulation has little effect on fluctuation in the Hb distribution as shown in Figure 7 and Figure S3, so that the effects of the Hb self-averaging mechanism on fluctuation can be tested by systematically modulating the fused Hb proteins.

Gregor et al. [12] also discussed the possible resolution of the paradox of signal interpretation through the self-averaging diffusion of Hb molecules. In their proposed mechanism, however, the positive feedback due to the binding of Hb protein to the *hb* enhancer was the necessary condition: They assumed that the *hb* genes in *N*≈50 neighboring nuclei are activated synchronously with the positive feedback interaction of the diffusing Hb and *hb* enhancers, thereby the effective size of the target DNA region is enlarged from *a* to *aN*, which leads to the modification of the criterion of Equation 1 to be 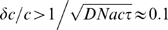. In the present simulations, however, we have not found an evidence of synchronized gene switching in multiple nuclei, so that this “self-averaging of input to the feedback loop” proposed in Ref.12 is not necessary to resolve the paradox of signal interpretation. The paradox was resolved even in models without the positive feedback, which clearly shows that the simpler assumption of “self-averaging of output” is sufficient to resolve the paradox. In *Drosophila* embryos, molecules other than Bcd or Hb may also affect the *hb* gene activity [18],[46]. The present results showed that assumption of the other input molecules is not necessary but the Bcd input and Hb output are sufficient to explain the stable, precise, and reproducible profiles of Bcd and Hb molecules at least in the anterior to middle regions of embryo.

Several different measures have been used to evaluate the accuracy of the Hb distribution [6], [9]–[12],[17],[36],[47],[48]. In this paper, we analyzed “preciseness” [12] of the Hb distribution by evaluating the smallness of fluctuation in each embryo, and “reproducibility” [6],[10],[12] by evaluating the smallness of fluctuation among multiple embryos. Other than these measures, “sharpness” [9],[36], and “robustness” [17],[47],[48] have been used to evaluate the accuracy. Our results showed that the sharp boundary of the Hb distribution is realized when *D*_h_ is within an optimal range: *D*_h_ should be smaller than 0.3 µm^2^/s to keep the average slope of the Hb boundary steep and *D*_h_ should be nonzero to suppress fluctuation around the average. In this way, the small fluctuation, or preciseness is a necessary condition for the sharpness. The present simulation showed that input of multiple kinds of transcriptional factors to *hb* is not necessary for the sharp Hb distribution. An experimental observation which is consistent with this result is the expression pattern of the reporter gene embedded into the zygotic genome [9]: The expressed pattern of the reporter gene which contains binding sites only for Bcd had a sharp boundary in embryos, which indicates that the other transcription factors than Bcd are not necessary for sharpness. This result is also consistent with the theoretical investigation: By solving the coupled equations of diffusion and reactions of Bialek and others [30]–[32], we can see that the physical limit of Equation 1 is not changed when the *hb* enhancer receives multiple input molecules. Thus, the multiple input molecules do not by themselves resolve the paradox of signal interpretation but the mechanism of self-averaging diffusion is necessary for preciseness and hence for sharpness of the Hb distribution. Interactions among multiple genes, however, should be important for the Hb distribution to be robust against variations of conditions. Comparison between the observed and simulated data has suggested that the feedback regulations among gap genes are necessary for the robust Hb distribution [47],[48]. This “canalization” mechanism, therefore, should be a necessary condition for robustness but our analyses suggest that the canalization mechanism by itself is not sufficient to assure preciseness and sharpness.

We emphasize that the system described by the three-dimensional model is not in a steady state, so that the absolute values of concentrations at each position vary during nuclear cycles. Effects of non-steadiness were also focused on by Bergmann et al. [15],[49]. Since the number of nuclei is small in the earlier stage of development, the fluctuation allowed to distinguish adjacent nuclei is about 50% in nuclear cycle 9 and 30% in nuclear cycle 11 by assuming an even distribution of nuclei during all nuclear cycles. Bergmann et al [49] suggested that the fate of each nucleus is determined as early as in nuclear cycle 9. Such pre-steady state decoding should tolerate the large fluctuation because of the large spatial distance between nuclei. Our simulation data showed that the system is not in the steady state all through nuclear cycles as pointed out by Bergmann et al, but the fluctuation in the simulated IO relation was larger than 30% in nuclear cycle 11 when *D*_h_ = 0. The pre-steady state decoding, therefore, does not resolve the paradox of signal interpretation by itself, but the slow diffusion of Hb is necessary for the precise decoding.

The mechanism of diffusion of Bcd remains as an unsolved problem. From the experimental data of the fluorescence relaxation of Bcd-eGFP, diffusion constant was estimated to be *D*_b_<1 µm^2^/s [11], but *D*_b_ = 10–20 µm^2^/s was suggested by monitoring diffusion of inert molecules in embryo [10]. One possible explanation of this discrepancy is based on the difference of environment in these measurements: The fluorescence relaxation giving small *D*_b_ was measured at the surface layer of embryo. Diffusion should be slow in such measurement because the complex and dense cytoskeletal structures in cortical layer trap the diffusing molecules [50],[51]. *D*_b_≈10–20 µm^2^/s, on the other hand, was a result of diffusion through the unstructured core of embryo. The fluorescence relaxation in less structured unfertilized embryo, however, also showed *D*_b_<1 µm^2^/s, which casts doubts on such simple interpretation based on the difference of environment [11]. Another possible explanation is based on the notion of anomalous diffusion. Particles diffusing through medium of crowded obstacles should show the diffusion as 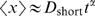 for the short distance, and 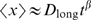 for the long distance with *α*<*β* [52]. If both 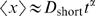 and 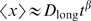 are fitted by 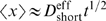 and 
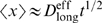
, the effective diffusion constant, 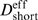, should become much smaller than 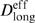, and hence it is possible to assume 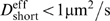 and 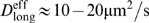. We can also expect that the cross-over distance between these two behaviors and other parameters such as 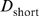 and 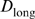 depend on the densities of cytoskeletal obstacles and take different values in cortical layer and core of embryos. Also possible is the mechanism that the cytoplasmic flow [14],[53] enhances transport of molecules and increases the effective value of *D*_b_ for the long spatio-temporal scale. Either type of the long-lived coherent flow [14] or the randomized flow should accelerate the movement of Bcd in embryo. Assumptions on values of diffusion constant in our models have not yet been checked from the microscopic viewpoint of the diffusion process, and further theoretical and experimental investigations are necessary to define diffusion constants on a sound basis.

The recent experimental report suggested that *bcd* mRNA is not strictly localized at the anterior pole but forms a gradient by the mechanism of quasi-random active transport through a nonpolar microtublular network in cortex of the embryo [41]. If this is the case, Bcd can form the gradient by following its mRNA gradient with the relatively small diffusion constant, so that the assumption of the anomalous diffusion of Bcd may not be necessary. Test of this mechanism of dictation of the *bcd* mRNA gradient is possible with the present one- and three-dimensional models by changing the definition of sites which can synthesize Bcd and adjusting diffusion constants of Bcd. With such modifications, the mechanism of Bcd gradient formation will be altered in the model but we expect that the main results of the paper, such as the stable Bcd distribution through the rapid transportation of Bcd into nuclei and the precise and reproducible Hb distribution through resolution of the paradox, will not be changed because these results should be independent of whether the Bcd gradient is formed by following the *bcd* mRNA gradient or it is formed by the localized synthesis and diffusion.

Another important problem is the scaling of distributions of developmental factors in embryo [54]. Several dipteran species have different size of embryos varying up to five fold, in which the distribution of Bcd scales with the length of embryo [10],[17],[34],[55],[56]. A possible mechanism of this scaling is the domination of degradation of Bcd at nuclei to determine the Bcd profile [10],[11]. Because the positional distribution of nuclei scales with the length of embryo, one might consider that this assumption leads to the scaling of Bcd distribution. We examined this hypothesis with our three-dimensional model, but the results indicated that the Bcd distribution does not scale even when nuclei distribution scales (data not shown). To solve this problem, the assumption of the larger rate of trapping to the nuclei might be needed [57], or the scaling of transport or diffusion mechanism of Bcd or *bcd* mRNA should be necessary. Examination of these hypotheses is left for future investigations.

## Methods

### 

#### Stochastic simulations

In both one- and three-dimensional models, stochastic simulations were performed with the Gillespie algorithm [58]. In each step in simulation with the Gillespie algorithm, one molecular process has to be picked out efficiently from a large number of diffusion and reaction processes defined in the model. Such efficient picking out was realized by sorting a large number of processes with the optimized direct method [59].

#### A guideline for parameterization

Most of the standard parameter values were determined by consulting the relevant experimental observation, and several parameters such as *J*, *g*(3), *h*_b_ and *h*_h_ in the one-dimensional model or *J*(*m*) and *Q*(*m*) in the three-dimensional model were calibrated to reproduce the observed averaged values of distributions of Bcd and Hb. The calculated results with this model, therefore, were derived by using the experimental data for the averaged values of distributions, but the experimental data for fluctuations of distributions were not used in the definition of the model or in determination of parameter values. The main objective of the model was not to show the consistency of the model to explain the observed average values of distributions of Bcd and Hb, but to show that the model provides consistent data on precision to read out the Bcd gradient.

#### Standard parameters in the one-dimensional model

Standard parameters used in the one-dimensional model are summarized in Table 1. These standard parameter values were determined by following the guideline mentioned above. To determine the standard value of *D*_b_ in Table 1, however, there remains a fundamental problem: When inert molecules whose molecular weight is comparable with Bcd were injected into embryo, the diffusion constant of those molecules obtained from the measurement for an hour was *D*_b_∼10–20 µm^2^/s [10]. This value of *D*_b_ can well explain the observed exponential gradient of Bcd in embryo [10]. When the diffusion constant was measured by the relaxation of fluorescence strength in the Bcd-eGFP distribution at the cortical cytoplasm for a minute, on the other hand, the obtained value was *D*_b_<1 µm^2^/s [11], which is far different from the value observed from the long spatio-temporal measurement. A possible explanation of this discrepancy is that Bcd diffuses anomalously because Bcd is frequently trapped by structures in cytoplasm during its diffusion [50],[51]. This anomalous diffusion may make *D*_b_ dependent on the distance *l* to be measured: For *l*>10 µm, *D*_b_ should be as large as 10–20 µm^2^/s, but for *l*<1 µm, *D*_b_ should be small enough to be less than 1 µm^2^/s. Another possible explanation is that the cytoplasmic flow in embryo [14],[53] effectively enhances the diffusion over long distance. Though either of these hypotheses has not yet been confirmed, we here rely on such possibilities and use *D*_b_ = 10 µm^2^/s as a standard parameter to model the cytosolic diffusion process.

Since 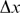 s varied in every simulation run, *V*_r_ is also varied as 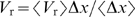 and the other volume dependent parameters are 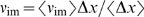, 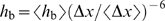, and 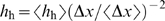. 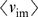 and 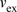 in Table 1 are related to each other by 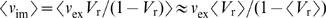. We consider that 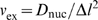, where 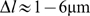 is a distance inside the nucleus, so that we use 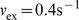 in Table 1. Diffusion constant of Hb in cytoplasm is assumed to be small because the observed data suggests that Hb diffuses for much smaller distance than Bcd [36], and hence we assume that *D*_h_ is as small as *D*_nuc_. We use *D*_h_ = 0.1 µm^2^/s as a standard parameter.

Bcd is stable in cytoplasm but could be degraded in nucleus during the interphase period, so that we assume that the lifetime of Bcd is similar to or longer than the duration of the interphase. Hb may also have the same order of lifetime. We use *k*_b_ = 7.7×10^−4^ s^−1^ = 1/21.6 min (half-life period of 15 min) and *k*_h_ = 3.9×10^−4^ s^−1^ in Table 1. The rate of synthesis of Bcd at the anterior pole *J* was adjusted to fit the calculated value of the Bcd concentration to the observed data. Considering that the diameter of nucleus is about 6.5 µm, *V*_nuc_ is around 144 µm^3^. The observed concentration of Bcd in nuclei shows a peak of 55 nM near the anterior pole and decays to 8 nM at around the middle point of the AP axis and becomes almost 0 at around the posterior pole [12], which correspond to 4700 molecules/nucleus at the anterior part, 700 molecules/nucleus at the middle part and almost 0 at the posterior part. In Table 1, *J* was chosen to fit the calculated *N*_Bcd_(*i*) to these values. For *N*_burst_, we use a typical value *N*_burst_ = 10 found in the single-molecule observation of a gene switch [60]. The synthesis rate of Hb becomes very low when Bcd does not bind to the *hb* enhancer [40], so that *g*(0) and *g*(1) have to be much smaller than *g*(2) or *g*(3). In Table 1 we assume *g*(0)/*g*(3) = *g*(1)/*g*(3) = 10^−5^. The experimental data showed that in the embryo which lacks ability of Hb to bind the *hb* enhancer, the concentration of Hb at the anterior region is reduced to ∼20% of that in the wild type [36],[40], so that we assume *g*(2)/*g*(3) = 0.2 in the standard parameterization. As *g*(3)*N*_burst_/*k*_b_ should give an estimation of *N*_Hb_(*i*) around the nucleus of the state *S*(*i*) = 3, we determined *g*(3) by adjusting the calculated results of *N*_Hb_(*i*) at the anterior part to have the same order of magnitude as *N*_Bcd_(*i* = 1).

The rate of unbinding Bcd or Hb from the *hb* enhancer can be of order of the duration of the interphase, so that we determine *f*_b_ and *f*_h_ to be 1/3 min≈5×10^−3^ s^−1^. The binding affinity of Bcd and Hb to the *hb* enhancer is determined by *f*_b_/*h*_b_ and *f*_h_/*h*_h_, respectively. Parameters *h*_b_ and *h*_h_, therefore, determine which nuclei should be *S*(*i*) = 2 or 3, and hence determine the region where *hb* actively expresses. We adjusted the values of *h*_b_ and *h*_h_ to fit the location of the sharp increase in the calculated *N*_Hb_(*i*) to the observed threshold position of *x*/*L*∼48% [12].

#### Standard parameters in the three-dimensional model

Standard parameter values are summarized in Table 2. Diffusion constants in the three-dimensional model depend on types of starting or arriving sites, which are explained in Figure 2C. In cortical sites, for example, microtubules or other skeletal structures are developed, which should trap molecules and slower the diffusive movement of molecules, and hence diffusion constant between cortical sites is set to be smaller than that between core sites. Since the cytoskeltal structure in cortical sites develops as embryo develops, Bcd should become more easily trapped to such structure in the later stage of nuclear cycles, which should decrease the diffusion constant *D*_Bcd, cortical-core_ in Figure 8C. We represent this tendency by introducing a decreasing factor *Q*(*m*) as a function of the nuclear cycle *m* as defined in Table 2. *Q*(*m*) was determined so as to reproduce the observed increase of concentration of Bcd at the cortical layer (Figure 6D of Ref.11). This gradually decreasing diffusion constant prevented Bcd from escaping into core sites to accumulate in the cortical layer. Values of other parameter in Table 2 were chosen according to the same guiding principle as in Table 1, but we make several comments on some specific features of parameters in the three-dimensional model: We assume that Bcd is actively transported into nuclear, so that the rate constant for transport of Bcd into the nuclear sites are larger than that for transporting Bcd to the outside of the nuclear sites in Table 2. Bcd and Hb are degraded with the rate of 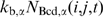 and 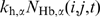 in cortical or core sites. In the nuclear sites, the degradation rates are 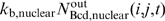 and 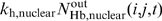 at the outside of the interaction volume, and 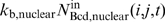 and 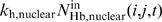 at the inside of the interaction volume. Here, 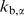 and 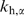 are assumed to be small in cortical or core sites but large in nuclear sites. At the core sites in the slice *i* = 1, Bcd is generated with the rate *J*(*m*) as in the one-dimensional model. Since the observed production rate of Bcd increases as nuclear cycles proceed [42], we assume that *J*(*m*) is an increasing function of *m*. The value of *J*(14) is adjusted so that the calculated results reproduce the observed concentration profile of Bcd at nuclear cycle 14.

The *hb* enhancer state in the three-dimensional model is *S*(*i*, *j*, *t*) = 0–3. Also assumed is that Hb is synthesized at around nuclei, i.e. in the nuclear site with the rate *g*(*S*(*i*, *j*, *t*)). Values of *g*(*S*(*i*, *j*, *t*)) can be determined in the same way as Equation 7 or Equation 8. In Table 2, g(3) was adjusted so that the calculated number of Hb molecules becomes of same order as that of Bcd molecules.

## Supporting Information

Text S1 One and Three-Dimensional Models.
(0.28 MB PDF)

Figure S1 Noise in the Input/Output Relation in the One-Dimensional Model with Varied Frequencies of the State Change of the *hb* Enhancer. *σ*_h_(*i*)/max[<*N*_h_(*i*)>] is plotted in the two dimensional plane of the relative rates of gene switching, *ω*_b_ = *f*_b_/*k*_b_ and *ω*_h_ = *f*_h_/*k*_h_. *V*_r_ = 10^−2.5^ is used. Other parameters are set to have standard values.
(0.12 MB PDF)

Figure S2 Input/Output Relations and Their Noise in the Three-Dimensional Model with Varied Parameters. Parameterization is changed by choosing one parameter from Table 2 to be varied from its standard value. For one parameterization, lines of 10 simulated runs are calculated as in each panel of Figure 11 and then these 10 lines are averaged to obtain one curve. Thus calculated averaged curves for different parameterizations are superposed. (Top row) Mean input/output relations. (Middle row) The standard deviation of input/output relations. (Bottom row) Translation of the data of Middle row into lines of equivalent input noise. (Left) The relative size of the interaction volume is varied as *V*_r_ = 10^−1^ (navy), 10^−1.5^ (dark green), 10^−2^ (light green), 10^−2.5^ (blue, standard value), 10^−2.9^ (orange), 10^−3^ (magenta), and 10^−3.5^ (red). (Second left) The dissociation rate of Bcd from the *hb* enhancer is varied as *ω*_b_ = *f*_b_/*k*_b, nuclear_ = 10^4^ (dark green), 10^3^ (light green), 10^2^ (blue, standard value), 10 (orange), 1 (magenta), and 10^−1^ (red). (Middle) The dissociation rate of Hb from the *hb* enhancer is varied as *ω*_h_ = *f*_h_/*k*_h, nuclear_ = 10^4^ (dark green), 10^3^ (light green), 10^2^ (blue, standard value), 10 (orange), 1 (magenta), and 10^−1^ (red). (Second Right) The number of Hb molecules synthesized in a burst is varied as *N*_burst_ = 100 (dark green), 50 (light green), 10 (blue, standard value), and 1 (red). The frequency of bursting, *g*(*S*), is also varied to keep *g*(*S*)*N*_burst_ constant. (Right) The diffusion constant of Hb is varied as *D*_h_ = 5 µm^2^/s (navy), 1 µm^2^/s (dark green), 0.5 µm^2^/s (light green), 0.25 µm^2^/s (light blue), 0.1 µm^2^/s (blue, standard value), 0.05 µm^2^/s (orange), 0.01 µm^2^/s (magenta), and 0 (red).
(0.26 MB PDF)

Figure S3 Input/Output Relations and Their Noise in the Three-Dimensional Model with Varied Parameters for the Case of No Feedback Regulation of *hb*. Parameterization is changed by choosing one parameter from Table 2 to be varied from its standard value. For one parameterization, lines of 10 simulated runs are calculated and then these 10 lines are averaged to obtain one curve. Thus calculated averaged curves for different parameterizations are superposed. The same plot as in Figure S2 but with *h*_h_ = 0.
(0.26 MB PDF)

Figure S4 Side-View of Simulated Embryos. Snapshots of the *hb* expression pattern at *t* = 95 min, which is in the interphase of nuclear cycle 12, are shown by highlighting nuclear sites of large γ with white dots. (A) *D*_h_ = 0.1 µm^2^/s, and (B) *D*_h_ = 0. Snapshots of the distribution pattern of Hb molecule are shown by green dots (C) *D*_h_ = 0.1 µm^2^/s at the same instance as in A, and (D) *D*_h_ = 0 at the same instance as in B.
(0.72 MB PDF)

Figure S5 Import and Export Dynamics of Nuclear Bcd. Snapshots of temporal changes of Bcd concentration at the slice *i* = 5 of the three-dimensional model. Bcd concentration is expressed by green shaded color. Nuclear cycle 13 starts from 103 min 00 sec and lasts until 119 min 00 sec. Bcd molecules start to accumulate in nuclei at the onset of the interphase (103 min 00 sec), and quickly flow out of nuclei at the onset of mitosis in nuclear cycle 14 (119 min 00 sec).
(0.17 MB PDF)

Figure S6 Development of Hb Profiles During Nuclear Cycle 14. (A) Numbers of Hb molecules averaged over 10 simulation runs, <*N*_h, nuclear_(*i*,100,*t*)>, are shown at every one minute during *t* = 126–143 min in nuclear cycle 14 as functions of *x*/*L* = *i*Δ*x*/*L*. The *j* = 100th sites are on the surface of the cylinder. (B) Position of *x*_1/2_/*L* = *i*_half_Δ*x*/*L* is plotted for *t* = 126–143 min in nuclear cycle 14 (blue line) Red dots are the experimental data of *x*_1/2_/*L* (Figure 2C of Ref.36). (C) Angle of slope of <*N*_h, nuclear_(*i*_half_,100,*t*)> is plotted at every one minute for *t* = 126–143 min in nuclear cycle 14 with the standard parameterization (blue dots) and with the parametrization of *h*_h_ = 0 (green dots). The lack of positive feedback with *h*_h_ = 0 makes the angle slightly smaller. Red dots are the experimental data of angle at *x*_1/2_/*L* (Figure 2D of Ref.36).
(0.17 MB PDF)

Video S1 Temporal Change of Distributions of Bcd and Hb in the Three-Dimensional Model. Concentrations of Bcd and Hb at the *i* = 5th slice are shown in the green shaded color. The simulated 80-minute process from nuclear cycle 8 to nuclear cycle 14 is shown in one-minute video run. (Left) Bcd concentration, and (Right) Hb concentration.
(0.89 MB WMV)
