# Supplementary material for: Stable, Precise, and Reproducible Patterning of Bicoid and Hunchback Molecules in the Early Drosophila Embryo
Source: PLoS Comput Biol. 2009 Aug 28;5(8):e1000486. doi: 10.1371/journal.pcbi.1000486 (PMC2720536; doi:10.1371/journal.pcbi.1000486)
Supplement: Text S1 — One and Three-Dimensional Models. (0.28 MB PDF) [file pcbi.1000486.s001.pdf]

Supporting Information, Text S1

Stable, Precise, and Reproducible Patterning of Bicoid and Hunchback Molecules in the early *Drosophila* embryo

Yurie Okabe-Oho[1], Hiroki Murakami[1], Suguru Oho[2]
and Masaki Sasai* [1,3,4]
[1]Department of Computational Science and Engineering,
Nagoya University, Nagoya 464-8603, Japan,
[2]Department of Environmental Engineering and Architecture,
Nagoya University, Nagoya 464-8601, Japan,
[3]Department of Applied Physics,
Nagoya University, Nagoya 464-8603, Japan,
and
[4]School of Computational Sciences,
Korea Institute for Advanced Study, Seoul 130-012, Korea

*sasai@nuap.nagoya-u.ac.jp

One-Dimensional Model of *Drosophila* Embryo

The problem of precise readout of the Bcd gradient is analyzed by developing a one-dimensional model of embryo. The model consists of 100 nuclei placed along the one-dimensional anterior-posterior (AP) axis. These nuclei stay static during the simulation. The system of length L is composed of 100 sites each of which has the length of $\Delta x = L/100$. Each site contains a nucleus, and numbers of Bcd and Hb molecules in the ith site are denoted by $N_{\mathrm{Bcd}}(i)$ and $N_{\mathrm{Hb}}(i)$, respectively.

Binding/unbinding of Bcd and Hb to/from the *hb* enhancer: $N_{\mathrm{Bcd}}(i)$ is divided into the part outside the interaction volume $N^{\mathrm{out}}_{\mathrm{Bcd}}(i)$, and the part inside the interaction volume $N^{\mathrm{in}}_{\mathrm{Bcd}}(i) + N^{\mathrm{bound}}_{\mathrm{Bcd}}(i)$, where $N^{\mathrm{in}}_{\mathrm{Bcd}}(i)$ is the number of Bcd molecules that remain in the interaction volume without binding to the *hb* enhancer, and $N^{\mathrm{bound}}_{\mathrm{Bcd}}(i)$ is the number of Bcd molecules that are bound to the *hb* enhancer. $N_{\mathrm{Hb}}(i)$ is also divided in

the same way, so that

$$N_{\mathrm{Bcd}}(i) = N_{\mathrm{Bcd}}^{\mathrm{out}}(i) + N_{\mathrm{Bcd}}^{\mathrm{in}}(i) + N_{\mathrm{Bcd}}^{\mathrm{bound}}(i),$$

$$N_{\mathrm{Hb}}(i) = N_{\mathrm{Hb}}^{\mathrm{out}}(i) + N_{\mathrm{Hb}}^{\mathrm{in}}(i) + N_{\mathrm{Hb}}^{\mathrm{bound}}(i). \qquad \text{(S1)}$$

We consider that only the molecules in the interaction volume can bind to the *hb* enhancer. Due to the intra-nuclear diffusion, molecules are stochastically transferred into the interaction volume as

$$N_{\mathrm{Bcd}}^{\mathrm{out}}(i) \to N_{\mathrm{Bcd}}^{\mathrm{out}}(i) - 1, \text{ and } N_{\mathrm{Bcd}}^{\mathrm{in}}(i) \to N_{\mathrm{Bcd}}^{\mathrm{in}}(i) + 1, \qquad \text{(S2a)}$$

$$N_{\mathrm{Hb}}^{\mathrm{out}}(i) \to N_{\mathrm{Hb}}^{\mathrm{out}}(i) - 1, \text{ and } N_{\mathrm{Hb}}^{\mathrm{in}}(i) \to N_{\mathrm{Hb}}^{\mathrm{in}}(i) + 1. \qquad \text{(S2b)}$$

In the simulation, processes of Equation S2 are stochastically called with the rate $v_{\mathrm{im,b}} N_{\mathrm{Bcd}}^{\mathrm{out}}(i)$ for Equation S2a and with the rate $v_{\mathrm{im,h}} N_{\mathrm{Hb}}^{\mathrm{out}}(i)$ for Equation S2b. The reverse processes are export of molecules from the interaction volume to the outside,

$$N_{\mathrm{Bcd}}^{\mathrm{out}}(i) \to N_{\mathrm{Bcd}}^{\mathrm{out}}(i) + 1, \text{ and } N_{\mathrm{Bcd}}^{\mathrm{in}}(i) \to N_{\mathrm{Bcd}}^{\mathrm{in}}(i) - 1, \qquad \text{(S3a)}$$

$$N_{\mathrm{Hb}}^{\mathrm{out}}(i) \to N_{\mathrm{Hb}}^{\mathrm{out}}(i) + 1, \text{ and } N_{\mathrm{Hb}}^{\mathrm{in}}(i) \to N_{\mathrm{Hb}}^{\mathrm{in}}(i) - 1, \qquad \text{(S3b)}$$

with the rate $v_{\mathrm{ex,b}} N_{\mathrm{Bcd}}^{\mathrm{in}}(i)$ for Equation S3a and with the rate $v_{\mathrm{ex,h}} N_{\mathrm{Hb}}^{\mathrm{in}}(i)$ for Equation S3b. As we assume that intra-nuclear diffusion constants of Bcd and Hb are same, we set $v_{\mathrm{im,b}} = v_{\mathrm{im,h}} = v_{\mathrm{im}}$ and $v_{\mathrm{ex,b}} = v_{\mathrm{ex,h}} = v_{\mathrm{ex}}$. We write the volume of a nucleus as V_{nuc}, so that

$$V_{\mathrm{r}} = V_{\mathrm{int}} / V_{\mathrm{nuc}}, \qquad \text{(S4)}$$

represents the relative size of the interaction volume. When processes of Equation S2a

and Equation S3a are balanced to sustain the steady state, we have $v_{\mathrm{im}}\overline{N_{\mathrm{Bcd}}^{\mathrm{out}}(i)} = v_{\mathrm{ex}}\overline{N_{\mathrm{Bcd}}^{\mathrm{in}}(i)}$, where the overbar represents average over the long enough time. Since there is no physical difference between inside and outside of the interaction volume, the average concentration of Bcd molecules does not depend on whether it is inside or outside of the interaction volume, so that we have $V_{\mathrm{r}} = \overline{N_{\mathrm{Bcd}}^{\mathrm{in}}(i)} \big/ \overline{N_{\mathrm{Bcd}}(i)}$. By neglecting the contribution from $N_{\mathrm{Bcd}}^{\mathrm{bound}}(i)$, these relations lead to the condition,

$$v_{\mathrm{im}} = v_{\mathrm{ex}} V_{\mathrm{r}}/(1 - V_{\mathrm{r}}). \qquad \text{(S5)}$$

In the standard parameterization, we have $V_{\mathrm{r}} << 1$, so that Equation S5 gives $v_{\mathrm{im}} << v_{\mathrm{ex}}$, which leads to $\overline{N_{\mathrm{Bcd}}^{\mathrm{out}}(i)} >> \overline{N_{\mathrm{Bcd}}^{\mathrm{in}}(i)}$ and $\overline{N_{\mathrm{Hb}}^{\mathrm{out}}(i)} >> \overline{N_{\mathrm{Hb}}^{\mathrm{in}}(i)}$. This smallness of $\overline{N_{\mathrm{Bcd}}^{\mathrm{in}}(i)}$ should bring about a large fluctuation in $N_{\mathrm{Bcd}}^{\mathrm{in}}(i)$.

The *hb* enhancer is assumed to stay in one of the following four states; the state with no bound Bcd or Hb ($S(i)$ = 0), the state with two bound Hb molecules and with no bound Bcd ($S(i)$ = 1), the state with six bound Bcd molecules and with no bound Hb ($S(i)$ = 2), and the state with six bound Bcd and two bound Hb molecules ($S(i)$ = 3). We consider the cooperative binding process of Bcd as

$$N_{\mathrm{Bcd}}^{\mathrm{in}}(i) \to N_{\mathrm{Bcd}}^{\mathrm{in}}(i) - 6, \text{ and } N_{\mathrm{Bcd}}^{\mathrm{bound}}(i) \to N_{\mathrm{Bcd}}^{\mathrm{bound}}(i) + 6,$$
$$S(i) = 0 \to S(i) = 2 \text{ or } S(i) = 1 \to S(i) = 3, \qquad \text{(S6)}$$

with the rate $\mathscr{H}_{b} = h_{\mathrm{b}} V_{\mathrm{r}}^{-6} \prod_{k=0}^{5}\left(N_{\mathrm{Bcd}}^{\mathrm{in}}(i) - k\right)$ and the cooperative binding process of Hb as

$$N_{\mathrm{Hb}}^{\mathrm{in}}(i) \to N_{\mathrm{Hb}}^{\mathrm{in}}(i) - 2, \text{ and } N_{\mathrm{Hb}}^{\mathrm{bound}}(i) \to N_{\mathrm{Hb}}^{\mathrm{bound}}(i) + 2,$$
$$S(i) = 0 \to S(i) = 1 \text{ or } S(i) = 2 \to S(i) = 3, \qquad \text{(S7)}$$

with the rate $\mathscr{H}_{\mathrm{h}} = h_{\mathrm{h}} V_{\mathrm{r}}^{-2} N_{\mathrm{Hb}}^{\mathrm{in}}(i)\left(N_{\mathrm{Hb}}^{\mathrm{in}}(i) - 1\right)$. Due to the large fluctuation of $N_{\mathrm{Bcd}}^{\mathrm{in}}(i)$ and $N_{\mathrm{Hb}}^{\mathrm{in}}(i)$, binding processes of Equation S6 and Equation S7 should bear intense noise. In this paper, embryos homozygous for the mutated *hb* allele, which codes for Hb having no-DNA binding affinity, are also simulated by putting $h_{\mathrm{h}} = 0$. For such mutant embryos, we only consider $S(i) = 0$ and $S(i) = 2$ as possible states of the *hb* enhancer.

Unbinding reactions are the reverse processes of Equations S6 and S7 as

$$N_{\mathrm{Bcd}}^{\mathrm{in}}(i) \to N_{\mathrm{Bcd}}^{\mathrm{in}}(i) + 6, \text{ and } N_{\mathrm{Bcd}}^{\mathrm{bound}}(i) \to N_{\mathrm{Bcd}}^{\mathrm{bound}}(i) - 6,$$
$$S(i) = 2 \to S(i) = 0 \text{ or } S(i) = 3 \to S(i) = 1, \qquad \text{(S8)}$$

with the rate f_{b}, and

$$N_{\mathrm{Hb}}^{\mathrm{in}}(i) \to N_{\mathrm{Hb}}^{\mathrm{in}}(i) + 2, \text{ and } N_{\mathrm{Hb}}^{\mathrm{bound}}(i) \to N_{\mathrm{Hb}}^{\mathrm{bound}}(i) - 2$$
$$S(i) = 1 \to S(i) = 0 \text{ or } S(i) = 3 \to S(i) = 2, \qquad \text{(S9)}$$

with the rate f_{h}. The ratio $f_{\mathrm{b}}/h_{\mathrm{b}}$ or $f_{\mathrm{h}}/h_{\mathrm{h}}$ determines the affinity of molecules to the *hb* enhancer site. We should also note that f_{b} and f_{h} can be used as measures of time scale of change in the *hb* enhancer state.

In the wild type diploid embryo, each nucleus has two *hb* enhancers at which binding/unbinding takes place independently of each other. For the sake of simplicity, however, we approximate this binding/unbinding by only considering a single effective *hb* enhancer in each nucleus. The effective rate constant to generate Hb from the single effective *hb* gene is set to be about twice larger than the rate constant to generate Hb from each individual zygotic gene. Such approximation may lead to errors in time correlation of the number of Hb molecules in a short time scale, but this simplification should not produce significant quantitative errors to examine the static or long-time fluctuations in Bcd and Hb distributions.

Diffusion, degradation, and synthesis of Bcd: Due to the diffusive movement in cytoplasm, Bcd molecules are transferred between adjacent sites as

$$N_{\rm Bcd}^{\rm out}(i) \to N_{\rm Bcd}^{\rm out}(i)-1, \text{and } N_{\rm Bcd}^{\rm out}(i+1) \to N_{\rm Bcd}^{\rm out}(i+1)+1 \ \text{ for } \ 1 \le i \le 99 ,$$

$$N_{\rm Bcd}^{\rm out}(i) \to N_{\rm Bcd}^{\rm out}(i)-1, \text{and } N_{\rm Bcd}^{\rm out}(i-1) \to N_{\rm Bcd}^{\rm out}(i-1)+1 \ \text{ for } \ 2 \le i \le 100 , \quad \text{(S10)}$$

with the rate $d_{\rm b}N_{\rm Bcd}(i)$, where $d_{\rm b}$ is proportional to the diffusion constant of Bcd, $D_{\rm b}$, as $d_{\rm b} = D_{\rm b}/\Delta x^2$. Bcd is degraded at each site as

$$N_{\rm Bcd}^{\rm out}(i) \to N_{\rm Bcd}^{\rm out}(i)-1 \quad \text{(S11a)}$$

with the rate $k_b N_{\rm Bcd}^{\rm out}(i)$, and

$$N_{\rm Bcd}^{\rm in}(i) \to N_{\rm Bcd}^{\rm in}(i)-1 \quad \text{(S11b)}$$

with the rate $k_b N_{\rm Bcd}^{\rm in}(i)$. Bcd is synthesized from the maternal mRNA distributed at around the anterior pole, which is simulated by assuming that Bcd is generated only at the site $i = 1$,

$$N_{\rm Bcd}^{\rm out}(1) \to N_{\rm Bcd}^{\rm out}(1)+1 \ , \quad \text{(S12)}$$

with the rate J.

Diffusion, degradation, and synthesis of Hb: Diffusion and degradation of Hb are treated in the same way as those of Bcd but with different rate constants:

$$N_{\rm Hb}^{\rm out}(i) \to N_{\rm Hb}^{\rm out}(i)-1, \text{and } N_{\rm Hb}^{\rm out}(i+1) \to N_{\rm Hb}^{\rm out}(i+1)+1 \ \text{ for } \ 1 \le i \le 99 ,$$

$$N_{\rm Hb}^{\rm out}(i) \to N_{\rm Hb}^{\rm out}(i)-1, \text{and } N_{\rm Hb}^{\rm out}(i-1) \to N_{\rm Hb}^{\rm out}(i-1)+1 \ \text{ for } \ 2 \le i \le 100 , \quad \text{(S13)}$$

with the rate $d_{\rm h}N_{\rm Hb}(i)$, where $d_{\rm h}$ is proportional to the diffusion constant $D_{\rm h}$ as $d_{\rm h} = D_{\rm h}/\Delta x^2$. Hb is degraded at each site as

$$N_{\text{Hb}}^{\text{out}}(i) \to N_{\text{Hb}}^{\text{out}}(i) - 1 \quad \text{(S14a)}$$

with the rate $k_h N_{\text{Hb}}^{\text{out}}(i)$, and

$$N_{\text{Hb}}^{\text{in}}(i) \to N_{\text{Hb}}^{\text{in}}(i) - 1 \quad \text{(S14b)}$$

with the rate $k_h N_{\text{Hb}}^{\text{in}}(i)$.

Reactions involved in transcription and translation processes are coarse-grained and described as a combined single reaction step. Then, the process to synthesize Hb around the ith nucleus is

$$N_{\text{Hb}}^{\text{out}}(i) \to N_{\text{Hb}}^{\text{out}}(i) + N_{\text{burst}}, \quad \text{(S15)}$$

where N_{burst} is the number of Hb molecules synthesized from a short-lived *hb* mRNA, representing the bursting production of Hb. The bursting production with N_{burst} >>1 can be a source of intrinsic noise of *hb* expression. We consider that Equation S15 is executed with the rate $g(S(i))$, where $S(i)$ =0-3 is the state of the *hb* enhancer in the ith site. As suggested in the experimental data [1, 2], binding of Hb and binding of Bcd cooperatively activate synthesis of Hb, and hence we assume

$$g(0) \le g(1) << g(2) < g(3). \quad \text{(S16)}$$

For a mutant of loosing binding affinity of Hb with $h_{\text{h}} = 0$, we only consider the case of $S = 0$ and $S = 2$, so that

$$g(0) << g(2). \quad \text{(S17)}$$

The scheme of Bcd-Hb cooperation of Equation S16 implies that Hb activates expression of *hb*, which constitutes a positive feedback loop of Hb production. In the scheme of Equation S17, on the other hand, the positive feedback loop is absent.

Three-Dimensional Model of *Drosophila* Embryo

Effects of dynamically changing nuclei are examined by developing the three-dimensional model of embryo. An embryo is represented by a cylinder of length $L \approx 490\mu m$ extending along the AP axis. The cylinder is composed of 70 plates each of which has thickness of $\Delta x = L/70$. Each plate has an approximately circular shape of radius 90μm, and consists of 625 hexagonal sites whose side length is $\beta \approx 4\mu m$. The system, therefore, consists of 70X625=43750 sites. Starting from t = 0 at the time of oviposition, the dynamical change of distribution of Bcd and Hb is followed with the stochastic simulation until t = 144min at the interphase of nuclear cycle 14. This simulation is repeated 10 times by varying β and Δx around averages $\langle \beta \rangle = 4.04\mu m$ and $\langle \Delta x \rangle = 7\mu m$ and with deviations of $\delta\beta / \langle \beta \rangle = 4.1\%$ and $\delta\Delta x / \langle \Delta x \rangle = 4.1\%$ to simulate the observed fluctuation of 4.1% in size of embryo [3].

The system has three types of sites; cortical, core, and nuclear sites. Each nuclear site contains a single nucleus and other types of sites do not contain nucleus. Reactions and diffusions defined at sites depend on types of sites. Position of site is designated by (i, j), where i is the number of slice from i = 1 at the anterior slice to i = 70 at the posterior slice. j = 1–625 designates the position of site in the ith slice. The numbers of Bcd and Hb in each site at time t are denoted by $N_{\mathrm{Bcd},\alpha}(i,j,t)$ and $N_{\mathrm{Hb},\alpha}(i,j,t)$, where α = cortical, core, or nuclear specifies the type of site.

Rules of mitoses and growth of cortical layer: Nuclei undergo nuclear cycles, increase in number, and change their locations in embryo. One nuclear cycle consists of the interphase period and the mitotic period. During the interphase period, nucleus is stable, which is described by a nuclear site in the model. During the mitotic period, nuclear membrane is dissolved and nuclear sites are turned into cortical or core sites. After the mitosis, nuclear membrane is rebuilt to enter into the next nuclear cycle. In the next nuclear cycle, the number of nuclei is doubled. In *Drosophila* embryo, it was observed that somatic nuclei undergo nearly synchronous nuclear divisions as a syncytium [4]. We treat these nuclear cycles as synchronized events in the model. At t = 0, one nuclear site is defined at around the center of embryo and nuclear cycle 1 is initiated. See Box S1 for further details of the rules.

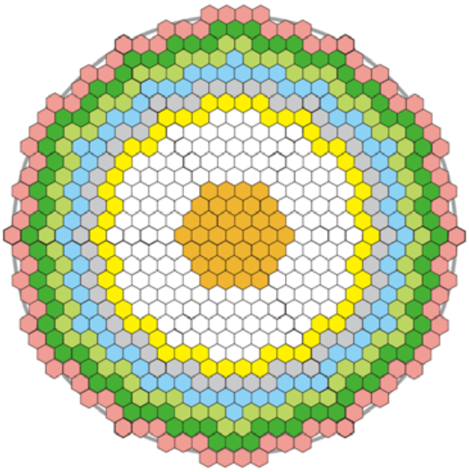

Box S1: Rules of mitoses and growth of cortical layer. Positions of nuclear and cortical sites in each nuclear cycle are determined by the following rules (i)-(vii). During nuclear cycle 1-7, the system has 2^{n-1} nuclear sites. (i) In nuclear cycle 1-2, nuclear sites are placed randomly in the orange-colored region of slices i = 52-53. (ii) In nuclear cycle 3-7, nuclear sites are placed randomly in the orange-colored region or in white region of slices i = 14-56. (iii) In nuclear cycle 8, $N_p \leq 2^7$ sites are selected as nuclear sites with the average $< N_p >$ = 90 and the deviation δN_p = 45. N_p sites are placed randomly in the yellow-colored region of slices i = 10-60. During nuclear cycle 8-14, the system has $2^{n-8}N_p$ nuclear sites. (iv) In nuclear cycle 9, N_p nuclear sites in nuclear cycle 8 move to the light-green-colored region by shifting along the radius direction in the slice. Other N_p nuclear sites newly created in nuclear cycle 9 are placed randomly in the light-green-colored region of slices i = 5-65. (v) In nuclear cycle 10, $2N_p$ nuclear sites in nuclear cycle 9 move to the pink-colored region by shifting along the radius direction in the slice. Other $2N_p$ nuclear sites newly created in nuclear cycle 10 are placed randomly in the pink-colored region of slices i = 2-70. (vi) In nuclear cycle 11-14, the newly created nuclear sites are added in random positions in the pink-colored region of slices i = 2-70. (vii) Layers of cortical sites increase as nuclear cycles proceed [4]: During nuclear cycle 1-10, sites in the pink colored-region are cortical sites if they are not nuclear sites. Cortical sites are sites in the pink and dark-green-colored regions (nuclear cycle 11), in the pink, dark-green, and light-green colored regions (nuclear cycle 12), in the pink dark-green, light-green, and light-blue colored regions (nuclear cycle 13), and in the pink, dark-green, light-green, light-blue colored and gray regions (nuclear cycle 14).

Diffusion and reactions: Diffusion and reactions of Bcd and Hb defined in the three-dimensional model are the straightforward extension of those considered in the one-dimensional model. Bcd and Hb diffuse between adjacent lattice sites in the three-dimensional space as in the same way as in the one-dimensional model.

Reactions in the nuclear sites are same as in the one-dimensional model: Equations S1-S9 are assumed by using the variables $N^{\text{out}}_{\text{Bcd, nuclear}}(i,j,t)$, $N^{\text{in}}_{\text{Bcd, nuclear}}(i,j,t)$, $N^{\text{bound}}_{\text{Bcd, nuclear}}(i,j,t)$, $N^{\text{out}}_{\text{Hb, nuclear}}(i,j,t)$, $N^{\text{in}}_{\text{Hb, nuclear}}(i,j,t)$, and $N^{\text{bound}}_{\text{Hb, nuclear}}(i,j,t)$ instead of $N^{\text{out}}_{\text{Bcd}}(i)$, $N^{\text{in}}_{\text{Bcd}}(i)$, $N^{\text{bound}}_{\text{Bcd}}(i)$, $N^{\text{out}}_{\text{Hb}}(i)$, $N^{\text{in}}_{\text{Hb}}(i)$, and $N^{\text{bound}}_{\text{Hb}}(i)$ in the one-dimensional model. Those quantities are defined as

$$N_{\text{Bcd, nuclear}}(i,j,t) = N^{\text{out}}_{\text{Bcd, nuclear}}(i,j,t) + N^{\text{in}}_{\text{Bcd, nuclear}}(i,j,t) + N^{\text{bound}}_{\text{Bcd, nuclear}}(i,j,t),$$

$$N_{\text{Hb, nuclear}}(i,j,t) = N^{\text{out}}_{\text{Hb, nuclear}}(i,j,t) + N^{\text{in}}_{\text{Hb, nuclear}}(i,j,t) + N^{\text{bound}}_{\text{Hb, nuclear}}(i,j,t). \quad \text{(S18)}$$

In this way, effects of random reception of Bcd or Hb molecules at the *hb* enhancer are simulated by introducing the nanometer scale spatial region of interaction volume as in the one-dimensional model. V_{r} designates the ratio of interaction volume to the volume of a nucleus, and its smallness is the source of fluctuations in the *hb* expression.

References

1. Lopes FJP, Vieira FMC, Holloway DM, Bisch PM, Spirov AV (2008) Spatial bistability generates *hunchback* expression sharpness in the *Drosophila* embryo. PLoS Comput. Biol. 4: e1000184.
2. Simpson-Brose M, Treisman J, Desplan C (1994) Synergy between the Hunchback and Bicoid morphogens is required for anterior patterning in *Drosophila*. Cell 78: 855-865.
3. Gregor T, Tank DW, Wieschaus EF, Bialek W (2007) Probing the limits to positional information. Cell 130: 153-164.
4. Foe VE, Alberts BM (1983) Studies of nuclear and cytoplasmic behaviour during the five mitotic cycles that precede gastrulation in *Drosophila* embryogenesis. J. Cell Sci. 61: 31-70.
