# Supplementary figures and images for: Stable, Precise, and Reproducible Patterning of Bicoid and Hunchback Molecules in the Early Drosophila Embryo
Source: PLoS Comput Biol. 2009 Aug 28;5(8):e1000486. doi: 10.1371/journal.pcbi.1000486 (PMC2720536; doi:10.1371/journal.pcbi.1000486)

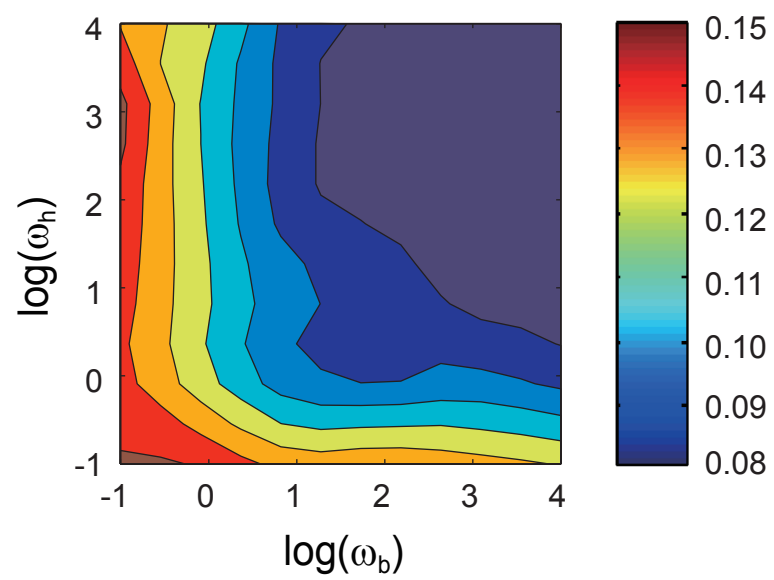

Supplement: Figure S1 — Noise in the Input/Output Relation in the One-Dimensional Model with Varied Frequencies of the State Change of the hb Enhancer. σ h(i)/max[] is plotted in the two dimensional plane of the relative rates of gene switching, ω b = f b/k b and ω h = f h/k h. V r = 10−2.5 is used. Other parameters are set to have standard values. (0.12 MB PDF) [file pcbi.1000486.s002.pdf]

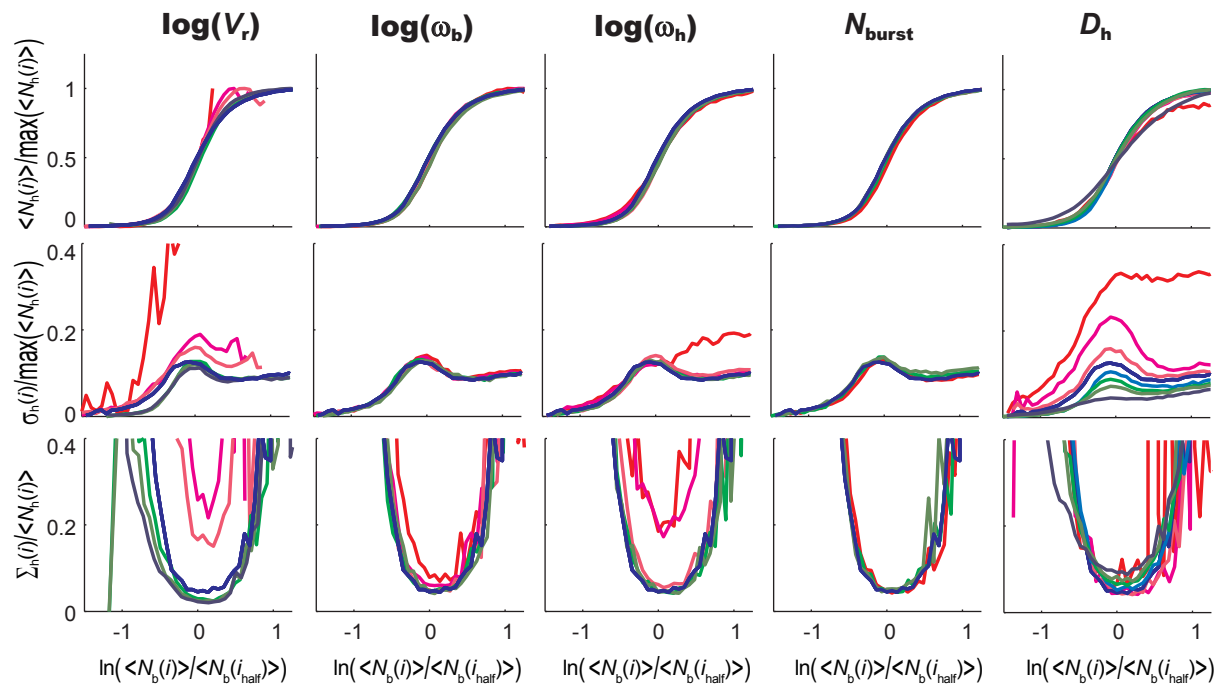

Supplement: Figure S2 — Input/Output Relations and Their Noise in the Three-Dimensional Model with Varied Parameters. Parameterization is changed by choosing one parameter from Table 2 to be varied from its standard value. For one parameterization, lines of 10 simulated runs are calculated as in each panel of Figure 11 and then these 10 lines are averaged to obtain one curve. Thus calculated averaged curves for different parameterizations are superposed. (Top row) Mean input/output relations. (Middle row) The standard deviation of input/output relations. (Bottom row) Translation of the data of Middle row into lines of equivalent input noise. (Left) The relative size of the interaction volume is varied as V r = 10−1 (navy), 10−1.5 (dark green), 10−2 (light green), 10−2.5 (blue, standard value), 10−2.9 (orange), 10−3 (magenta), and 10−3.5 (red). (Second left) The dissociation rate of Bcd from the hb enhancer is varied as ω b = f b/k b, nuclear = 104 (dark green), 103 (light green), 102 (blue, standard value), 10 (orange), 1 (magenta), and 10−1 (red). (Middle) The dissociation rate of Hb from the hb enhancer is varied as ω h = f h/k h, nuclear = 104 (dark green), 103 (light green), 102 (blue, standard value), 10 (orange), 1 (magenta), and 10−1 (red). (Second Right) The number of Hb molecules synthesized in a burst is varied as N burst = 100 (dark green), 50 (light green), 10 (blue, standard value), and 1 (red). The frequency of bursting, g(S), is also varied to keep g(S)N burst constant. (Right) The diffusion constant of Hb is varied as D h = 5 µm2/s (navy), 1 µm2/s (dark green), 0.5 µm2/s (light green), 0.25 µm2/s (light blue), 0.1 µm2/s (blue, standard value), 0.05 µm2/s (orange), 0.01 µm2/s (magenta), and 0 (red). (0.26 MB PDF) [file pcbi.1000486.s003.pdf]

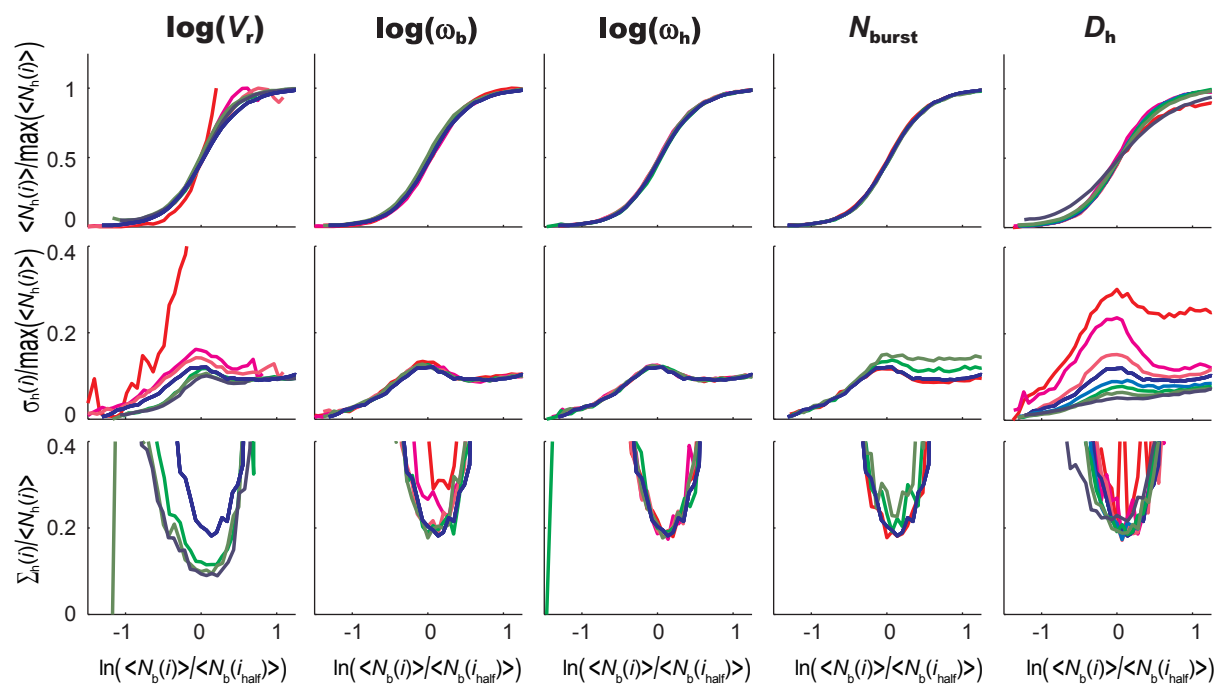

Supplement: Figure S3 — Input/Output Relations and Their Noise in the Three-Dimensional Model with Varied Parameters for the Case of No Feedback Regulation of hb. Parameterization is changed by choosing one parameter from Table 2 to be varied from its standard value. For one parameterization, lines of 10 simulated runs are calculated and then these 10 lines are averaged to obtain one curve. Thus calculated averaged curves for different parameterizations are superposed. The same plot as in Figure S2 but with h h = 0. (0.26 MB PDF) [file pcbi.1000486.s004.pdf]

**A**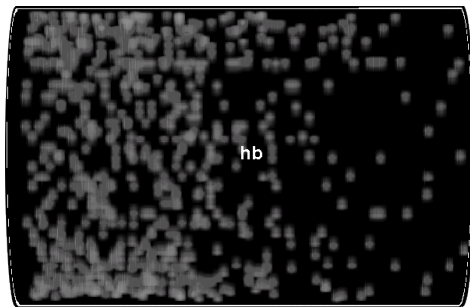**B**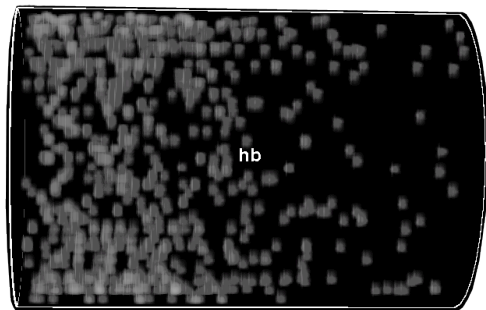**C**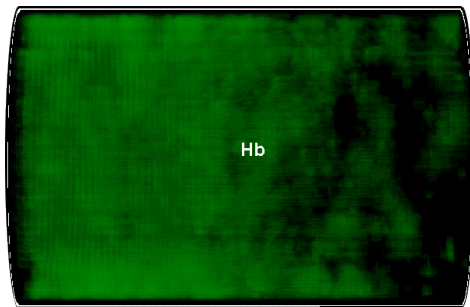**D**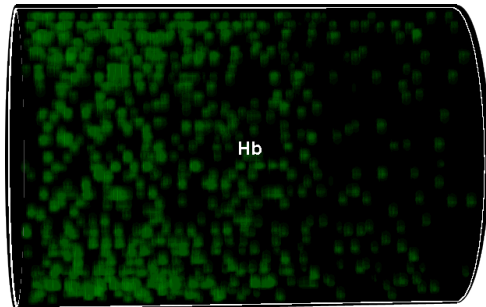

Supplement: Figure S4 — Side-View of Simulated Embryos. Snapshots of the hb expression pattern at t = 95 min, which is in the interphase of nuclear cycle 12, are shown by highlighting nuclear sites of large γ with white dots. (A) D h = 0.1 µm2/s, and (B) D h = 0. Snapshots of the distribution pattern of Hb molecule are shown by green dots (C) D h = 0.1 µm2/s at the same instance as in A, and (D) D h = 0 at the same instance as in B. (0.72 MB PDF) [file pcbi.1000486.s005.pdf]

102min 40sec

103min 00sec

103min 10sec

103min 20sec

103min 40sec

104min 50sec

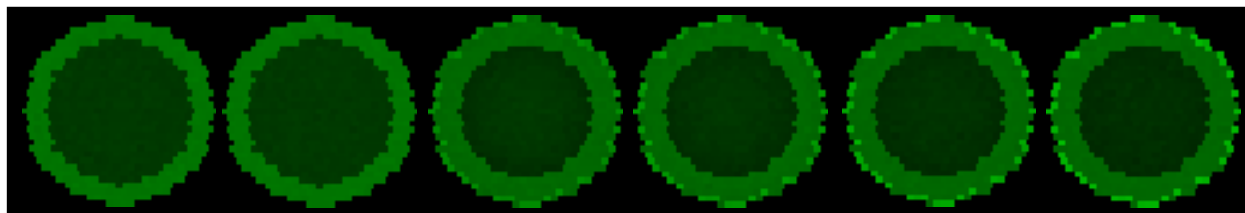

108min 50sec

114min 50sec

119min 00sec

119min 10sec

119min 20sec

119min 30sec

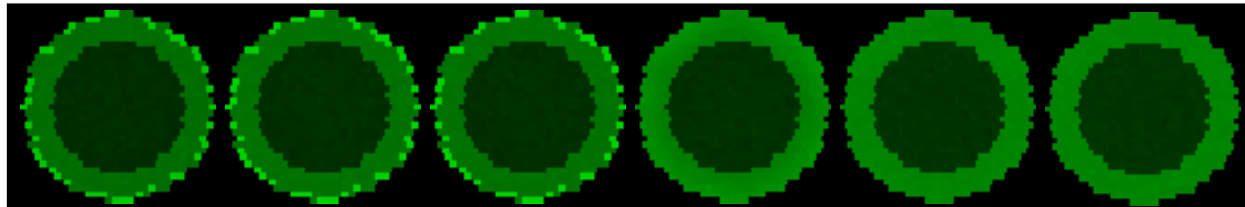

Supplement: Figure S5 — Import and Export Dynamics of Nuclear Bcd. Snapshots of temporal changes of Bcd concentration at the slice i = 5 of the three-dimensional model. Bcd concentration is expressed by green shaded color. Nuclear cycle 13 starts from 103 min 00 sec and lasts until 119 min 00 sec. Bcd molecules start to accumulate in nuclei at the onset of the interphase (103 min 00 sec), and quickly flow out of nuclei at the onset of mitosis in nuclear cycle 14 (119 min 00 sec). (0.17 MB PDF) [file pcbi.1000486.s006.pdf]

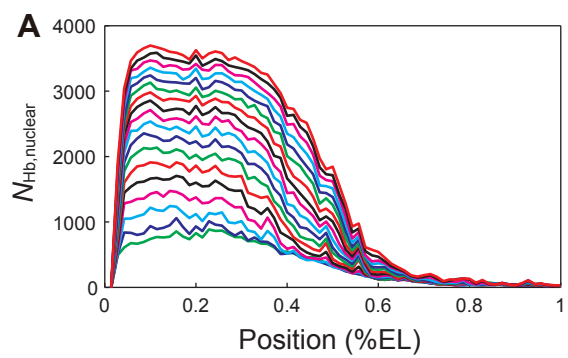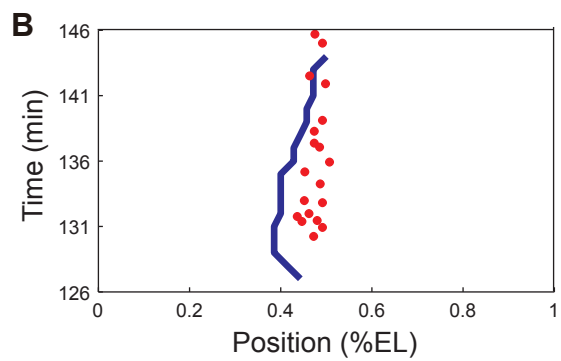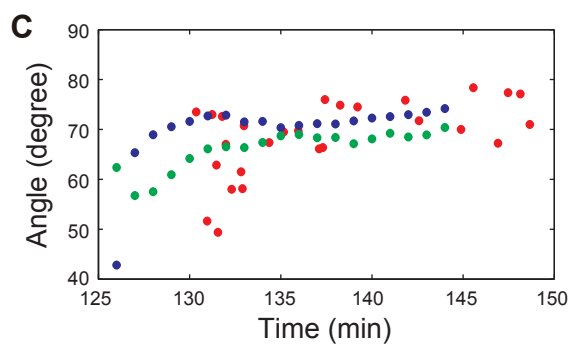

Supplement: Figure S6 — Development of Hb Profiles During Nuclear Cycle 14. (A) Numbers of Hb molecules averaged over 10 simulation runs, , are shown at every one minute during t = 126–143 min in nuclear cycle 14 as functions of x/L = iΔx/L. The j = 100th sites are on the surface of the cylinder. (B) Position of x 1/2/L = i halfΔx/L is plotted for t = 126–143 min in nuclear cycle 14 (blue line) Red dots are the experimental data of x 1/2/L (Figure 2C of Ref.36). (C) Angle of slope of is plotted at every one minute for t = 126–143 min in nuclear cycle 14 with the standard parameterization (blue dots) and with the parametrization of h h = 0 (green dots). The lack of positive feedback with h h = 0 makes the angle slightly smaller. Red dots are the experimental data of angle at x 1/2/L (Figure 2D of Ref.36). (0.17 MB PDF) [file pcbi.1000486.s007.pdf]
